# Cyclophilin A contributes to shikonin-induced glioma cell necroptosis and promotion of chromatinolysis

**DOI:** 10.1038/s41598-022-19066-y

**Published:** 2022-08-29

**Authors:** Xinyu Wang, Liwen Fan, Xuanzhong Wang, Tianfei Luo, Linlin Liu

**Affiliations:** 1 Department of Breast Surgery, Second Hospital of Jilin University, Changchun, China; 2 Department of Radiotherapy, China-Japan Union Hospital of Jilin University, Changchun, China; 3 Department of Neurosurgery, First Hospital of Jilin University, Changchun, China; 4 Department of Neurology, First Hospital of Jilin University, Changchun, China

**Keywords:** CNS cancer, Chemotherapy

## Abstract

Shikonin induces glioma cell death via necroptosis, a caspase-independent programmed cell death pathway that is chiefly regulated by receptor-interacting serine/threonine protein kinase1 (RIP1) and 3 (RIP3). Chromatinolysis is considered as one of the key events leading to cell death during necroptosis. It is usually accompanied with nuclear translocation of AIF and formation of γ-H2AX. Cyclophilin A (CypA) is reported to participate in the nuclear translocation of AIF during apoptosis. However, it remains unclear whether CypA contributes to necroptosis and regulation of chromatinolysis. In this study, our results revealed for the first time that shikonin promoted time-dependent CypA activation, which contributed to nuclear translocation of AIF and γ-H2AX formation. In vitro studies showed that knockdown of CypA by siRNA or inhibition of CypA by its specific inhibitor, cyclosporine A (CsA), not only significantly mitigated shikonin-induced glioma cell death, but also prevented chromatinolysis. Mechanistically, activated CypA targeted mitochondria and triggered mitochondrial superoxide overproduction, which then promoted AIF translocation from mitochondria into the nucleus by depolarizing the mitochondria and intensified the formation of γ-H2AX by promoting intracellular accumulation of ROS. Additionally, the CypA in the nucleus can form DNA degradation complexes with AIF and γ-H2AX, which also promote the execution of chromatinolysis. Thus, we demonstrate that CypA contributes to shikonin-induced glioma cell necroptosis and promotion of chromatinolysis.

## Introduction

Glioma is one of the most common primary malignant tumors of the nervous system with a high incidence rate. Treatments for glioma in clinic mainly include surgery combined with radiotherapy and chemotherapy^1^. However, the mean survival time of most glioma patients is less than one year^2^. Because glioma cells are anti-apoptotic, they are insensitive to radiotherapy and chemotherapy. Therefore, more effective treatment strategies are in urgent need of development.

Different from apoptosis, necroptosis is a caspase-independent mode of programmed cell death. Its biochemical features are similar with apoptosis, but it displays necrosis-like morphological features^3^. In the process of necroptosis, receptor interacting serine-threonine protein kinases1 (RIP1) is activated. Then the activated RIP1 interact with its downstream signal RIP3 through their RIP homotypic interaction motifs to form protein complexes called necrosomes. In addition, RIP3 can also be activated by self-phosphorylation^4^. The process of necroptosis is usually accompanied with cell swelling, cellular membrane disruption, DNA damage and energy depletion. DNA double strand breaks(DSBs) is the most serious form of DNA damage, it can be triggered by chemotherapeutic drugs, ionizing radiation and oxidative stress^5^ and lead to chromatinolysis and cell death^6,7^. In the process of DNA DSBs, DNA-dependent protein kinase catalytic subunit (DNA-PKcs) and ataxia telangiectasia mutated (ATM) are activated to promote the phosphorylation of histone variant H2AX to γ-H2AX^8^. Thus, γ-H2AX is generally considered as a marker of DNA DSBs^9,10^. Meanwhile, γ-H2AX can also recruit AIF in the mitochondria and Cyclophilin A (CypA) in the cell to the nucleus, forming a DNA degradation complex and promoting chomatinolysis^6,11,12^.Existing studies indicate that DNA DSBs is the key factor resulting chomatinolysis. However, the role of DNA DSBs in necroptosis remains elusive.

Shikonin is a kind of naphthpquinone that is extracted from *Lithospermum erythrorhizon*. It has been used for more than 2000 years for the treatment of infections, inflammation and bleeding diseases. Shikonin has been demonstrated to induce necroptosis in multiple myeloma and osteosarcoma cells. In our previous studies, we have demonstrated that shikonin can induce necroptosis in glioma cells, activate its downstream signaling factor MLKL by activating RIP1 and RIP3, and promote chromatinolysis by causing nuclear translocation of AIF and the formation of γ-H2AX^13^. At the same time, it has been reported that CypA is also involved in the nuclear translocation of AIF during apoptosis and can positively regulate the nuclear translocation of AIF, and promote chromatinolysis by combining with AIF and γ-H2AX to form the DNA degradation complex. In addition, existing evidences indicated that ROS contributed to necrosome complex formation and RIP1 and RIP3 activation during necroptosis. We also found in our previous study that mitochondrial superoxide regulated shikonin-induced activation of RIP1 and RIP3 in glioma^13^. However, it is unclear whether CypA is involved in shikonin-induced necroptosis and whether it is regulated by RIP1 and RIP3. Therefore, in this study, we used human and rat glioma cell lines and mice model of xenograft glioma to investigate the role of CypA in shikonin-induced chromatinolysis and the underlying mechanism.

## Materials and methods

### Reagents

Shikonin, Cyclosporine A (CsA), Nec-1, GSK872 and MnTBAP were purchased from selleckchem company (Houston, TX). Shikonin was dissolved in PBS to a storage concentration of 50 mmol/L. The primary antibodies against the following proteins RIP1, p-RIP1, RIP3, p-RIP3, Cyclophilin A, AIF, DKAPKcs, p-DNAPKcs, ATM, p-ATM, p-H2AX, H2A, NRF2, p-NRF2 antibodies were all purchased from Abcam company (Cambridge, MA). Anti-β-Actin antibody was from Santa Cruz Biotechnology (Santa Cruz, CA). Other reagents were purchased from Sigma (St. Louis, MO).

### Cell lines and culture

Human U87, U251, U373, SHG44 and rat C6 glioma cells were all obtained from Shanghai Institute of Cell Biology, Chinese Academy of Sciences (Shanghai, China). They were cultured in DMEM supplemented with 10% fetal bovine serum, 2 mmol/L glutamine, penicillin (100 U/mL) and streptomycin (100 μg/mL), and maintained at 37 ºC and 5% CO_2_ in a humid environment. Cells in the mid-log phase were used in the experiments.

### Cell viability and cell death assay

The cells were seeded onto 96-well microplate, cultured for 24 h and treated with target compounds at indicated concentrations. Cellular viability was assayed using an MTT assay and was expressed as a ratio to the absorbance value at 570 nm of the control cells. Cell death was assessed by using lactate dehydrogenase cytotoxicity assay kit according to the manufacturer’s instructions (Beyotime Biotech, Nanjing, China), the absorbance value of each sample was read at 490 nm, and cell death ratio was calculated by using the following formula: cell death ratio % = (A sample − A control/A max − A control) × 100. A sample: sample absorbance value; A control: the absorbance value of control group; A max: the absorbance value of positive group.

### Measurement of intracellular ROS and mitochondrial superoxide

Intracellular ROS was evaluated by using DCFH-DA (Beyotime Biotech, Nanjing, China) according to manufacturer’s instruction. The fluorescence was measured at an excitation wavelength of 485 nm and an emission wavelength 530 nm using a fluorescence spectrometer (HTS 7000, Perkin Elmer, Boston, MA). The ROS levels were expressed as arbitrary unit/mg protein, then as the percentage of control.

Mitochondrial superoxide was assayed by using MitoSOX red according to manufacturer’s description (Invitrogen, Eugene, OR). The red fluorescence density was measured at an excitation wavelength of 510 nm and an emission wavelength at 580 nm, and was expressed as a ratio to the fluorescence in control cells.

Other groups of cells were seeded onto a 6-well culture plate, stained with DCFH-DA or MitoSOX red as described above, and observed under fluorescence microscope (Olympus IX71, Tokyo, Japan).

### Mitochondrial membrane potential assays

Mitochondrial membrane potential was assayed by using JC-1 staining (Beyotime Biotech, Nanjing, China). The U87 and U251 cells treated with targets compounds were collected and stained with JC-1 according to manufacturer’s instruction. The collected cells were analyzed by flow cytometry (FACScan, Becton Dickinson, San Jose, CA).

Another group of U87 and U251 (4 × 10^5^ cells) glioma cells seeded onto a culture dish with a diameter of 3 cm were treated and stained with JC-1 as same as above, but observed under fluorescence microscope (Olympus IX71, Tokyo, Japan).

### Detection of DNA DSBs by neutral comet assay

Neutral comet assay was performed as described in our previous report^14^. The cells were suspended in 1 ml of low-melting agarose and 80 μL were deposited on comet slides prelayered with 1% regular agarose, covered with coverslips and allowed to gel at 4 °C for 10 min. Then, the coverslip was moved and the slide was covered with 80 μL low-melting agarose. Slides were immersed in lysing solution in dark at 4 °C for 1 h, washed for 10 min in TBE buffer, horizontally electrophoresed for 20 min at 25 V, and washed in 0.9% NaCl for 2 min. Cells were neutralized using 0.4 mol/L Tris (pH 7.5) and stained with ethidium bromide for 5 min. The slides were analyzed using a fluorescence microscope (Olympus IX71, Tokyo, Japan). The cell number with DNA comets and the DNA percent content in comet tail region were measured using ImageJ and Open Comet 1.3 software (three assays, each with about 100 cells analyzed).

### Transfection of small interfering RNA (SiRNA)

U87 (5 × 10^4^ cells/well) and U251(5 × 10^4^ cells/well) glioma cells were seeded onto a culture dish with a diameter of 10 cm. Transfection of siRNA was performed by using Lipofectamine 3000 (Invitrogen, USA) according to manufacturer’s instructions.

- RIP1 siRNA (5’-GCCAGCUGCUAAGUACCA ATT-3’),
- RIP3 siRNA (5′-UUCUCCGAACGUGUCACGUTT-3’),
- AIF siRNA (5′-GCAGUGGCAAGUUACUUAUTT-3′),
- CypA siRNA (5′-GCUCGCAGUAUCCUAGAAUTT-3’)

and scrambled SiRNA (5′-UUCUCCGAACGUGUCACGUTT-3′) were all purchased from GenePharma Company (Suzhou, China). After SiRNA transfection overnight, the cells were incubated with shikonin at indicated dosage for subsequent experiments.

### Gel Electrophoreses and western blotting

The collected glioma cells by centrifugation and the frozen xenografted glioma tissue were homogenized with a glass Pyrex micro homogenizer (20 strokes) in ice cold lysis buffer (Beyotime Biotech, Nanjing, China). Homogenates were centrifuged at 800 g for 10 min at 4 ºC to obtain the supernatant 1 and the pellet 1. The supernatant 1 was then centrifuged at 12,000 g for 10 min at 4 ºC to obtain supernatant 2 and pellet 2. The pellet 1 was nuclear fraction, supernatant 1 was cytoplasmic fraction, pellet 2 was mitochondrial fraction, and supernatant 2 was cytoplasmic fraction without mitochondria. The protein content was determined using Bio-Rad protein assay kit. After SDS electrophoresis and transfer to PVDF membranes, the membranes were blocked with 3% BSA in TBS for 2–4 h at room temperature, and then incubated overnight at 4 ºC with primary antibodies. Blots were cut prior to incubation with antibodies during blotting. After incubation with horseradish peroxidase-conjugated secondary antibody and washing the blots, immunoreactive proteins were visualized on a chemi-luminescence developer (ChemiScope 5300, Clinx Scicence Instrument Company, Shanghai).

### Extraction of genomic DNA and agarose gel electrophoresis

The cells were harvested by using 0.25% trypsin and collected by centrifugation for 10 min at 2000 rev/min. Then, the collected cells were incubated overnight at 55 °C with constant shaking in 200 μL of SDS lysis buffer to extract the genomic DNA. A volume of each sample equivalent to 10 μg of DNA was mixed with 6 × DNA loading dye and subjected to gel electrophoresis on a 1% agarose gel. DNA bands on the gel were visualized by UV transillumination and gel images were captured on the Gel Doc instrument (Bio-Rad Laboratories, Hercules, CA).

### Rat C6 tumor xenograft in mice

Twenty athymic BALB/c nude mice (aged 4 weeks, weight 20-22 g, from Beijing Vital River laboratory animal technology company, China) were housed in a specific pathogen-free environment under the condition of 12-h light/ 12-h dark cycle, free access to food and water. Principles of laboratory animal care were followed and all procedures were conducted according to the ARRIVE guidelines. This study was approved by the ethics committee of First Hospital of Jilin University (Changchun, China). A total of 1 × 10^7^ logarithmically growing C6 cells in 100 μL of PBS were subcutaneously injected into the right flank of each mouse. Therapeutic experiments were started when the tumor reached about 300 mm^3^ after 10 days. The mice were allocated to receive intraperitoneal injections of vehicle (n = 10/group), 2 mg/kg body weight shikonin in the same volume once two days for four times (n = 10/group). The tumor size was measured using a slide caliper, and the tumor volume was calculated using the formula: 0.5 × A × B2, in which A is the length of the tumor and B is the width. On the next day of the last treatment, the mice were euthanized by cervical dislocation. After being excised and weighed, the tumors were frozen immediately in liquid nitrogen for western blotting analysis.

### Gel Electrophoreses and western blotting

The cells seeded on a culture dish were fixed in ethanol, washed with PBS, and incubated with 1%Triton X-100 for 10 min. After the nonspecific antibody binding sites were blocked by 5% BSA, the cells were incubated with primary antibody against cyclophilin A (1:100), and then with in Cy3-conjugated goat anti-rabbit IgG (1:200) for 1 h at room temperature, followed by incubation with Heochst33258 for 30 min. Another group of cells were incubated with 100 nmol/L Mitotracker red ((Invitrogen company, Eugene, OR)) for 30 min at 37 °C before fixation in ethanol. After the nonspecific antibody binding sites were blocked, the cells were incubated with anti-BNIP3 (1:100) or anti-AIF antibody (1:100) followed by incubation in Alexa Fluor 488-conjugated goat anti-rabbit IgG (1:200) for 1 h and then with Heochst33258. Finally, all the cells were visualized under laser scanning confocal microscope (Olympus FV1000, Tokyo, Japan).

### Statistical analysis

All data represent at least 4 independent experiments and are expressed as mean ± SD. Statistical comparisons were made using One-way ANOVA. *p*-values of less than 0.05 were considered to represent statistical significance.

## Results

### Shikonin induced time-dependent cell death and chromatinolysis in glioma cells

In our previous study, we have demonstrated that shikonin inhibited the viabilities of glioma cells in a concentration-dependent manner, and the IC50 value of shikonin at incubation 3 h was about 6μ mol/L in C6 cells, 4μ mol/L in SHG44 cells and 6μ mol/L in U87, U251 and U373 cells^14–16^. Thus, we used these dosages of shikonin in the subsequent experiments.

**Figure 1:**
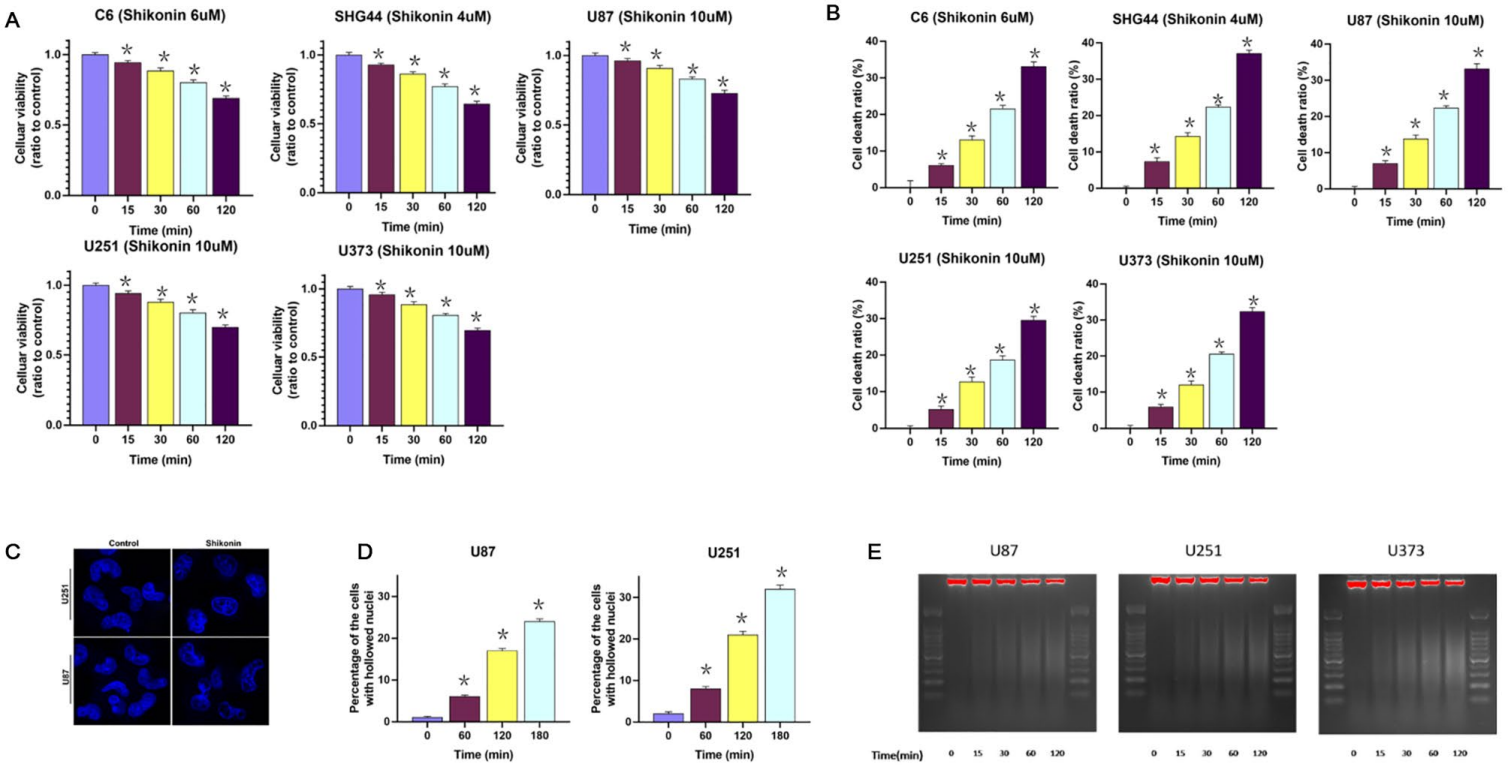
Shikonin induced time-dependent cell death and chromatinolysis in glioma cells. (**A**) MTT assay showed that shikonin induced time-dependent manner inhibition in the viabilities of C6, SHG44, U87, U251 and U373 glioma cells at incubation with IC50 dosages. (**B**) LDH release assay also showed that shikonin induced glioma cell death at IC50 dosages in a time-dependent manner. The values are expressed as mean ± SEM (n = 6 per group). *p < 0.01. (**C**) Fluorescence microcopy in combination with Hoechst 33,258 staining revealed that the nuclei in the U87 and U251 glioma cells became hollowed in shikonin-treated group cells compared with the ones in control. (**D**) The percentage of the hollowed nuclei to all the counted nuclei was increased significantly in a time-dependent manner after treated with shikonin. (**E**) Compared with the control group, the nucleus DNA of the shikonin treated U87, U251 and U373 cells showed continuous smear bands on the agarose gel. As the treatment time of shikonin extended from 15 to 120 min, the smear band became more obvious, which further confirmed the degradation of shikonin on nuclear DNA. The values are expressed as mean ± SEM (n = 5 per group). *p < 0.01.

Cellular viabilities were determined by using MTT assay. As shown in Fig. 1A, the cellular viabilities were decreased significantly in the glioma cells treated with shikonin at above-mentioned dosages in a time-dependent manner. Moreover, LDH release assay was performed to further confirm the effect of shikonin-induced glioma cells death. After incubation with shikonin at IC50 dosages for 30 min, 60 min, 120 min and 180 min, the cells death ratio were increased to 6.60 ± 1.50%, 13.05 ± 2.10%, 21.53 ± 2.00%, 33.05 ± 2.31% in C6 cell, 7.30 ± 2.08%, 14.23 ± 2.03%, 22.30 ± 1.65%, 37.09 ± 1.94% in SHG44 cell, 6.94 ± 1.83%, 13.79 ± 1.61%, 22.36 ± 1.61%, 33.14 ± 2.45% in U87 cell, 5.17 ± 1.96%, 12.63 ± 2.34%, 18.60 ± 2.19%, 29.50 ± 2.14% in U251 cell and 5.87 ± 1.79%, 11.94 ± 2.12%, 20.51 ± 1.65%, 32.34 ± 2.14% in U373 cell (Fig. 1B). Therefore, we demonstrated that shikonin not only inhibited the viabilities of glioma cells, but also induced glioma cell death in a time-dependent manner.

Since chromatinolysis is one of the events that ultimately lead to the cell death^17^, we used transmission electronic microscope to investigate shikonin-induced changes in chromatin in our previous study. As showed^13^, the nucleus of control group displayed smoothly outlines and contained clumps of heterochromatin. However, after treated with shikonin at IC50 dosages for 3 h, the nucleus of glioma cells became electron-lucent despite that the nuclear membrane was intact. This suggested that shikonin induce chromatin degradation in glioma cells.

Hoechst 33,258 is a kind of blue fluorescence dyes and often used to stain nuclear DNA. As revealed by laser scanning confocal microcopy, the nuclei stained with Hoechst 33,258 became hollowed in shikonin-treated group cells compared with the ones in control (Fig. 1C). Then the kinetics of shikonin-induced nuclear morphological changes in U87 cells and U251 cells were analyzed. The results showed that the percentage of the hollowed nuclei was increased significantly in a time-dependent manner after treated with shikonin (Fig. 1D). Furthermore, agarose gel electrophoresis is used to separate DNA fragmentation induced by different stresses. In contrast to apoptosis, the DNA showed continuous smear bands on agarose gel under necrotic condition. In order to further confirm that shikonin could induce chromatinolysis in glioma cells, nuclear DNA was extracted from U87, U251 and U373 glioma cells which were treated with shikonin at the dosage of IC50 values for the indicated time and electrophoresed on agarose gel. Compared with the control group, we found that the nucleus DNA of the shikonin treated cells showed continuous smear bands on the agarose gel (Fig. 1E). In addition, as the treatment time of shikonin extended from 15 to 120 min, the smear band became more obvious, which further confirmed the degradation of shikonin on nuclear DNA.

### CypA contributed to shikonin-induced chromatinolysis and glioma cell necroptosis

**Figure 2:**
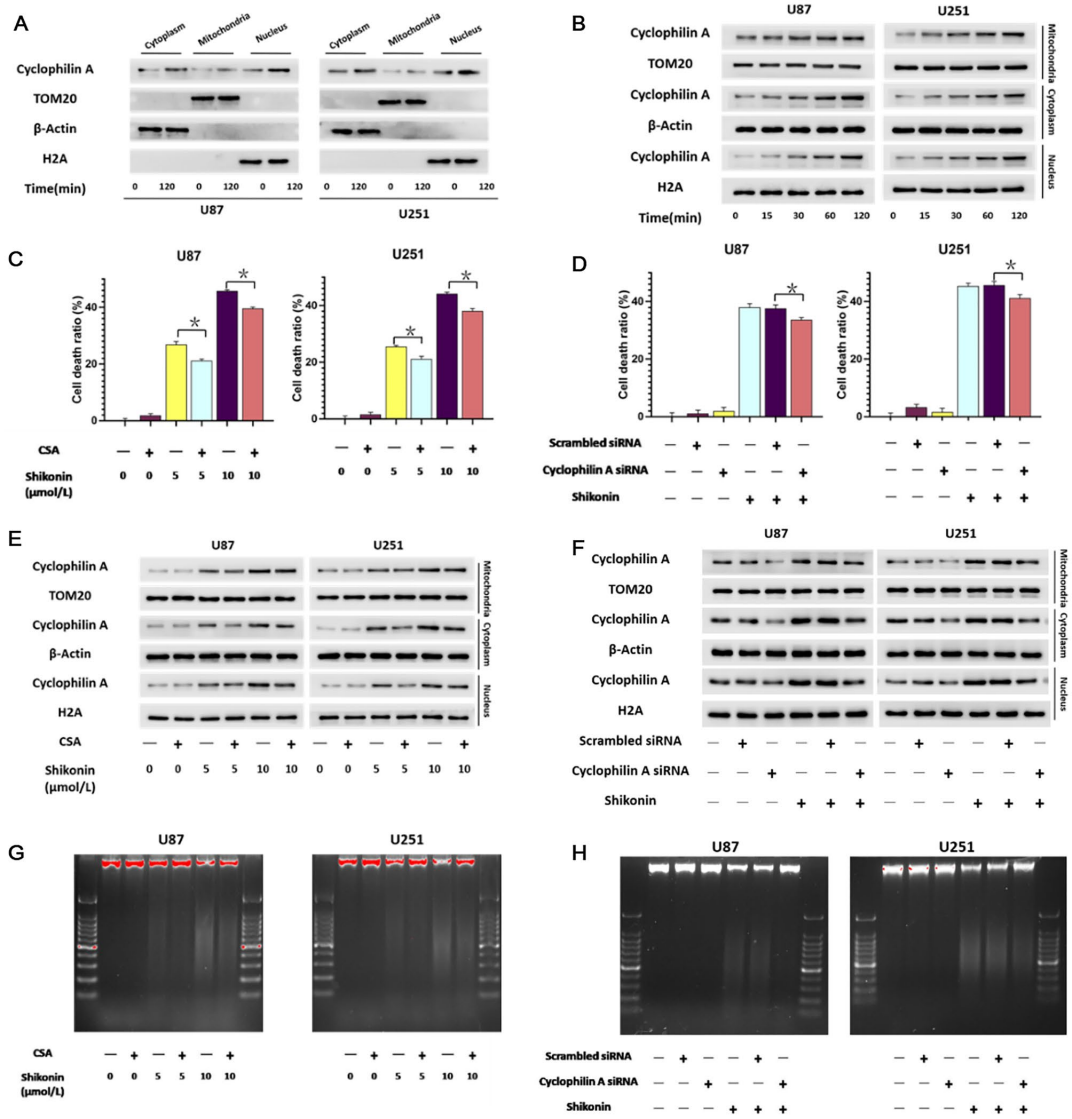
CypA contributed to shikonin-induced chromatinolysis and glioma cell necroptosis. (**A**) Western blotting revealed that shikonin induced time-dependent upregulation of CypA in U87 and U251 glioma cells. (**B**) Western blotting analysis showed that shikonin could increase CypA in cytoplasm, mitochondria and nucleus of glioma cells at the dosage of IC50 values. (**C**) LDH assay showed that CypA inhibitor CsA significantly attenuated the glioma cell death at incubation 3 h caused by shikonin at lower or higher concentrations. (**D**) LDH assay demonstrated that knockdown of CypA with siRNA could significantly inhibited the increase of glioma cell death induced by shikonin. (**E**) Western blotting showed that the up-regulation of CypA induced by shikonin was respectively inhibited by CsA in the U87 and U251 glioma cells. (**F**) Western blotting proved that CypA siRNA respectively suppressed the expressional up-regulation of CypA caused by shikonin. (**G**) Agarose gel electrophoresis showed that pretreatment with CsA abrogated shikonin-induced chromatinolysis in U87 and U251 glioma cells. (**H**) Agarose gel electrophoresis demonstrated as well that knockdown of CypA with siRNA prevented shikonin-induced chromatinolysis in U87 and U251 glioma cells. The values are expressed as mean ± SEM (n = 5 per group). *p < 0.01.

More and more evidences have confirmed that shikonin induced glioma cell necroptosis via activation of RIP1 and RIP3^14,18^, in which the role of CypA remains elusive. Western blotting was used to analyze the effect of shikonin on CypA of U87 and U251 glioma cells. As shown in Fig. 2A, compared with the control group, the protein levels of CypA in the cytoplasm, mitochondria and nucleus of U87 and U251 cells treated with shikonin were significantly increased. When the culture time was extended from 30 to 120 min, the protein level was significantly increased (Fig. 2B), indicating that the activation of CypA induced by shikonin was time-dependent. This indicated that shikonin induced CypA activation in a time-dependent manner.

To further clarify the role of CypA in shikonin-induced glioma cell death, specific inhibitor CsA and small RNA interference (siRNA) were introduced to inhibit the activation of CypA. The glioma cells were treated with CypA inhibitor CsA at 50 μmol/L for 1 h, and then incubated with shikonin for 3 h. LDH assay showed that CsA significantly prevented shikonin-induced glioma cell death (Fig. 2C). Meanwhile, western blotting showed that the up-regulated expression of CypA in the cytoplasm, mitochondria and nucleus were inhibited markedly when the cells were pretreated with CsA (Fig. 2E). Furthermore, we knocked down of CypA with siRNA to examine its effect on shikonin-induced glioma cell death in U87 and U251 cells. Consistently, LDH assay showed that knockdown of CypA could obvious inhibited shikonin-induced glioma cell death at incubation three hours (Fig. 2D). Western blotting also showed that knockdown of CypA suppressed the expressional up-regulation of CypA caused by shikonin (Fig. 2F). Therefore, all these results indicated that CypA contributed to shikonin-induced glioma cell necroptosis.

Notably, agarose gel electrophoresis of the nuclear DNA extracted from the cell treated with or without shikonin revealed that the smear bands of DNA induced by shikonin were alleviated obviously in the presence of CypA inhibitor CsA or when CypA was genetically knocked down with siRNA (Fig. 2G,H). Therefore, these data indicated that CypA contributed to shikonin-induced chromatinolysis and glioma cell necroptosis.

### CypA accounted for shikonin-induced mitochondria damage and nuclear translocation of AIF

**Figure 3:**
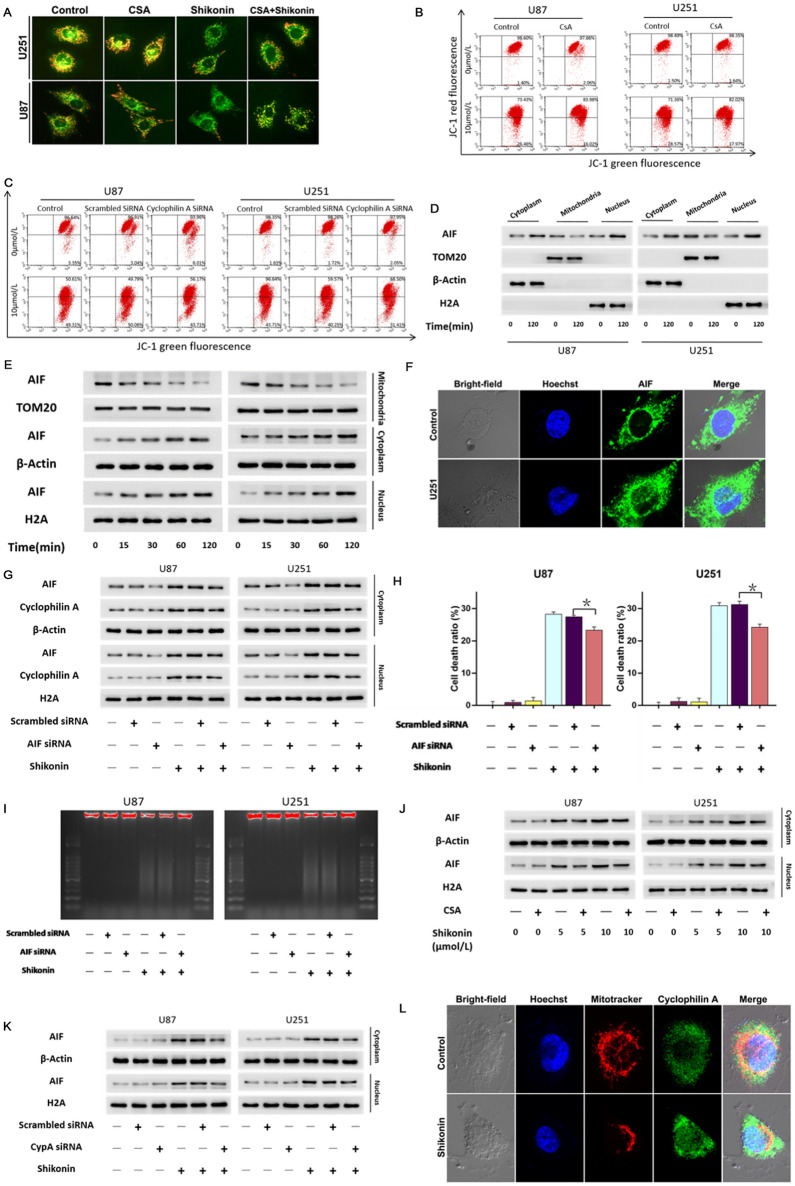
CypA accounted for shikonin-induced mitochondria damage and nuclear translocation of AIF. (**A**) The results of fluorescence microscopy combined with JC-1 staining showed that the green fluorescence of U87 and U251 cells increased significantly after treated with shikonin. These changes could be reversed by CypA inhibitor CsA. (**B**,**C**) Flow cytometry analysis with JC-1 staining showed as well that shikonin-induced depletion of mitochondrial membrane potential was obviously inhibited by CsA or CypA siRNA. (**D**) Western blotting analysis showed that AIF in mitochondria decreased, but AIF in cytoplasm and nucleus increased at the dosage of IC50 values. (**E**) Western blotting revealed that the change protein level of AIF induced by shikonin was in a time-dependent manner. (**F**) Laser scanning confocal microscopy showed that AIF was accumulated in the nucleus of the cells treated with shikonin compared with the control group. (**G**) Knockdown of AIF with siRNA prevented shikonin-induced improvement in nuclear AIF. (**H**) LDH release assay proved that knockdown of AIF prevented shikonin-induced death in U87 and U251 glioma cells. (**I**) Agarose gel electrophoresis of nuclear DNA showed that the DNA smear band treated with shikonin obviously alleviated in the knocked down AIF cells. (**J**,**K**) Western blotting analysis showed that shikonin-induced improvement of nuclear AIF levels was inhibited in the presence of CsA or CypA siRNA. (**L**) The representative confocal images showed that CypA (green) localized at mitochondria (red) and nuclear (blue) in the glioma cells treated with shikonin at the dosage of IC50 value for 2 h. The values are expressed as mean ± SEM (n = 5 per group). *p < 0.01.

Considering damaged mitochondrial played a crucial role in necroptosis, we thus speculated shikonin might induce mitochondria damage. Furthermore, mitochondrial membrane potential depletion is a sensitive indicator of mitochondrial damage. Thus, we used JC-1 staining to investigate whether shikonin treatment can lead to mitochondrial depolarization. JC-1 aggregates in healthy mitochondria and presents red fluorescence, but exists in the cytoplasm when mitochondrial membrane potential depletes and emits green fluorescence. Fluorescence microscopy revealed that compared with the control group, the green fluorescence of U87 and U251 cells increased significantly after treated with shikonin for 2 h. Moreover, CsA could significantly prevented shikonin-induced mitochondrial depolarization (Fig. 3A). Flow cytometry analysis proved as well that the green fluorescence increased obviously after the cells were treated with shikonin at either lower or higher dosage for 2.5 h. Consistently, inhibited CypA with inhibitor CsA or siRNA were obvious prevented shikonin-induced mitochondrial depolarization (Fig. 3B,C). These results indicated that CypA accounted for shikonin-induced mitochondria damage.

Since mitochondria are the normal position of AIF, the release of AIF from mitochondria is controlled by mitochondrial membrane potential^19^. AIF translocation from mitochondria to nuclei contributed to necrotic chromatinolysis^20^. Western blotting analysis showed that after shikonin treated U87 and U251 cells for 2 h, AIF in mitochondria decreased, but AIF in cytoplasm and nucleus increased (Fig. 3D). Moreover, the change of AIF protein level was time-dependent (Fig. 3E). In addition, laser scanning confocal microscopy showed that AIF was also accumulated in the nucleus of the cells treated with shikonin compared with the control group (Fig. 3F). This indicated that shikonin induced AIF release from mitochondrion and translocation in nucleus.

In order to investigate the effect of nuclear AIF in shikonin-induced glioma cells death, we introduced siRNA to inhibit AIF. Compared with the cells transfected with scrambled siRNA, the improvement of AIF induced by shikonin was significantly inhibited when AIF was knocked down by siRNA (Fig. 3G). LDH release experiment showed that AIF knockdown could prevent the cell death induced by shikonin (Fig. 3H). It is noteworthy that agarose gel electrophoresis of nuclear DNA showed that the DNA smear band treated with shikonin obviously alleviated in the knocked down AIF cells (Fig. 3I). It is suggested that AIF is involved in the chromatinolysis and glioma cell death induced by shikonin.

Then, we tested whether CypA could regulate the nuclear translocation of AIF by Western blotting analysis, and found that the inhibition of CypA with inhibitor CsA or knockdown CypA with siRNA could effectively inhibit the improvement of AIF induced by shikonin (Fig. 3J,K). Consistently, laser scanning confocal microscopy revealed that shikonin induced accumulation of CsA (green) on mitochondria (red) and nuclear (blue) (Fig. 3L). The results confirmed that CypA contributed to the AIF nuclear translocation induced by shikonin.

### CypA accounted for shikonin-induced DNA DSBs

**Figure 4:**
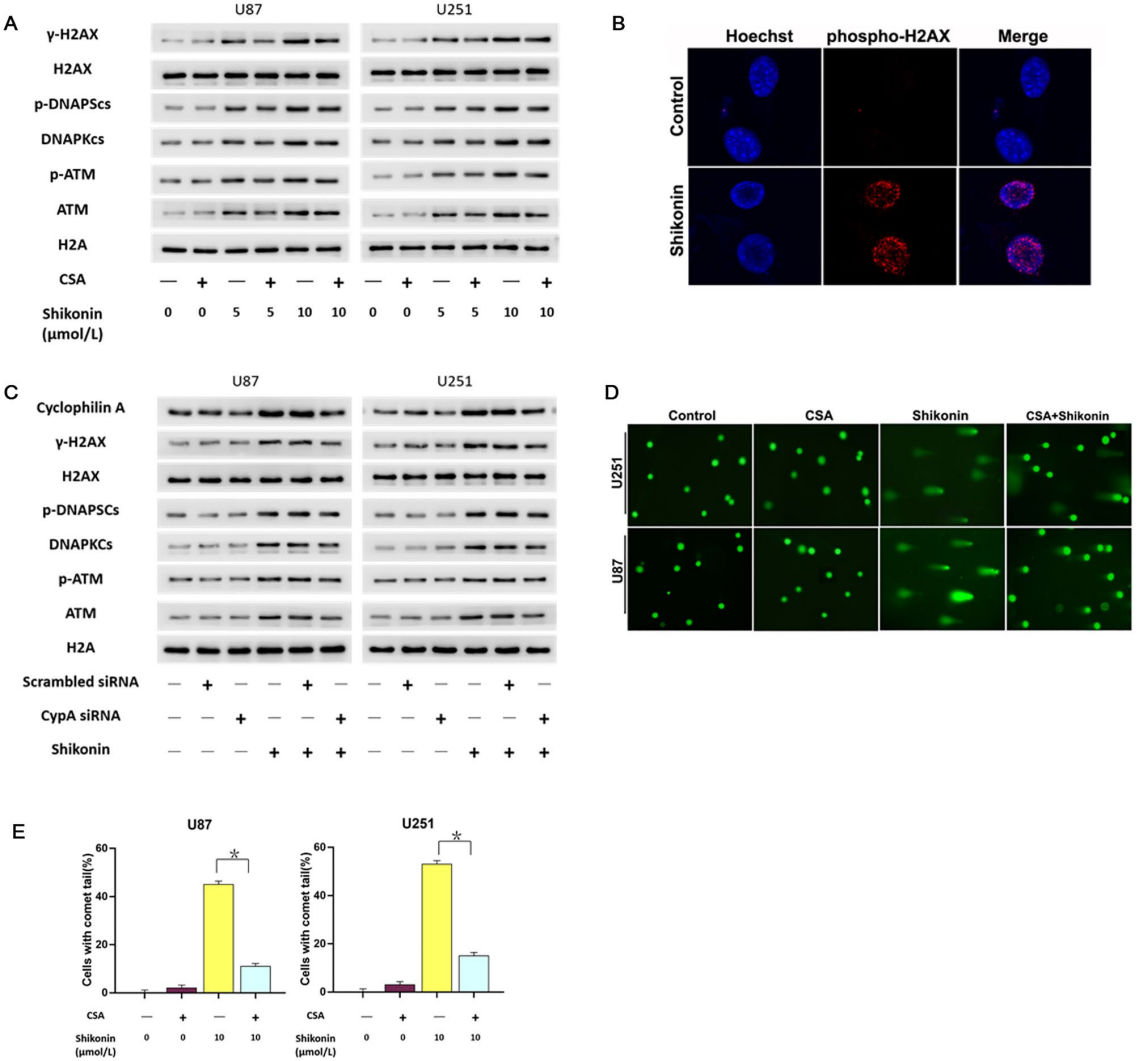
CypA accounted for shikonin-induced DNA DSBs. (**A**) Western blotting analysis showed that the protein level of γ-H2AX, p-ATM and p-DNAPKcs were improved significantly treated with shikonin at either lower or higher concentrations, which was prevented under CypA inhibitor CsA. (**B**) Representative images of confocal microscopy combined with immunochemical staining showed that mangy γ-H2AX foci formed in the nucleus of shikonin-treated cells. (**C**) Knockdown of CypA with siRNA prevented shikonin-induced phosphorylation of ATM and DNAPKcs and formation of γ-H2AX. (**D**) Neutral comet assay proved that compared with those in control group, shikonin-treated cells had longer comet tails which were obviously inhibited when the cells were pretreated with CypA inhibitor CsA. (**E**) Statistical analysis showed that CsA pretreatment could prevent the increase in the number of comet tail cells and the increase in the DNA content of comet tail induced by shikonin. The values are expressed as mean ± SEM (n = 5 per group). *p < 0.01.

Considering that DNA DSBs are the key step leading to chromatinolysis, we analyzed the change of γ-H2AX (phosphorylation-h2ax at ser139) induced by shikonin with Western blotting method, which is considered as a sensitive marker of DNA DSBs^21^. As western blotting analysis showed that the protein level of γ-H2AX was improved significantly treated with shikonin at either lower or higher concentrations (Fig. 4A). Furthermore, laser scanning confocal microscopy showed that there were multiple foci of γ-H2AX (red) in the nucleus (blue) treated with shikonin (Fig. 4B). Meanwhile, the phosphorylation levels of ataxia telangiectasis mutation (ATM) and DNA dependent protein kinase catalytic subunit (DNAPKcs) which are responsible for H2AX phosphorylation were both improved in shikonin treated cells (Fig. 4A,C). Western blotting also showed that inhibited the expression of CypA by inhibitor CsA or CypA siRNA could suppressed the expressional up-regulation of γ-H2AX, p-ATM and p-DNAPKcs induced by shikonin (Fig. 4A,C). Therefore, these data indicate that CypA promotes the formation of γ-H2AX induced by shikonin by phosphorylation of ATM or DNAPKcs.

Then, neutral comet assay which is often used to detect DNA DSBs proved as well that shikonin-treated cells had longer comet tails when compared with those in control group (Fig. 4D). Furthermore, the results of statistical analysis showed that shikonin treatment not only increased the number of comet tail cells, but also increased the DNA content of comet tail cells (Fig. 4E). But, the increases of the cells with comet tails and the improvement of DNA content in the comet tails induced by shikonin were both inhibited in the presence of CsA (Fig. 4D,E). It is suggested that CypA was involved in the regulation of shikonin-induced DNA DSBs in glioma cells.

### CypA increased intracellular ROS via causing overproduction of mitochondrial superoxide

**Figure 5:**
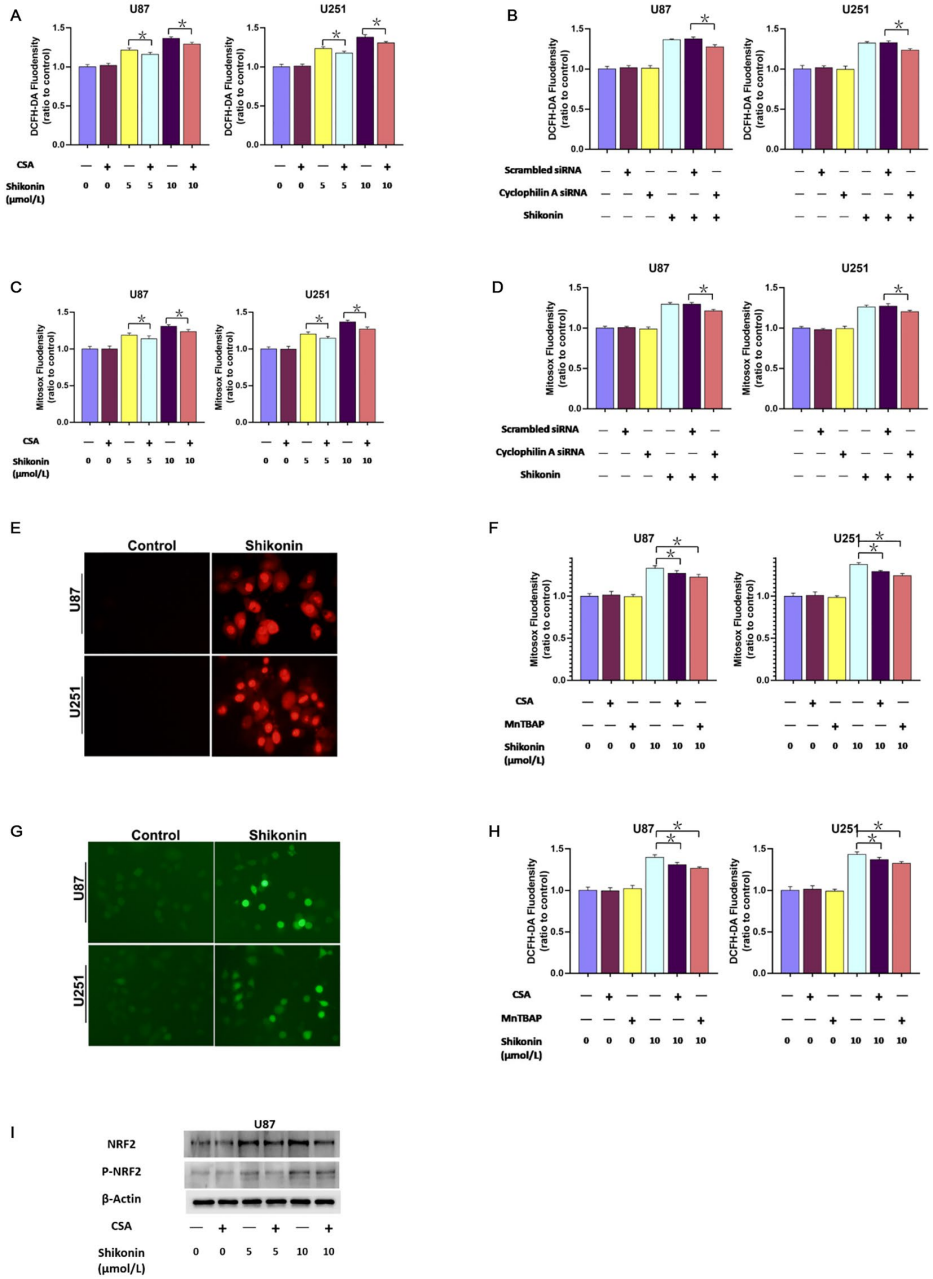
CypA increased intracellular ROS via causing overproduction of mitochondrial superoxide. (**A**–**D**) Shikonin could increase intracellular ROS and mitochondrial superoxide which can be alleviated by CypA inhibitor CsA and CypA siRNA. (**E**) Representative images of the glioma cells incubated with mitochondrial superoxide probe Mitosox red under fluorescence microscope (20 ×). Fluorescence microscopy showed that the red fluorescence of the cells treated with shikonin was much brighter than that of the cells in the control group. Pretreatment with 40 μmol/L MnTBAP for 1 h significantly prevented the generation of mitochondrial superoxide induced by shikonin. (**F**) Statistical analysis showed that CsA or MnTBAP pretreatment could inhibit the production of mitochondrial superoxide induced by shikonin. (**G**) Fluorescence microscopy showed that the green fluorescence of the cells treated with shikonin was much brighter than that of the cells in the control group. Pretreatment with 50 μmol/L CsA or 40 μmol/L MnTBAP for 1 h significantly inhibited the intracellular ROS induced by shikonin. (**H**) Statistical analysis showed that shikonin-induced abnormal increases of intracellular ROS could be inhibited in the presence of CsA and MnTBAP. The values are expressed as mean ± SEM (n = 5 per group). *p < 0.01.

Our previous report has confirmed that over-generated intracellular ROS is a crucial step contributing to DNA DSBs^14^. Meanwhile, mitochondria superoxide is a potent source of intracellular ROS. Therefore, we speculate that CypA plays an important role in mitochondrial oxidative stress. As it showed in Fig. 5A,C, shikonin could increase intracellular ROS and mitochondrial superoxide which can be alleviated by CypA inhibitor CsA. Consistently, knockdown CypA with siRNA not only inhibited shikonin-induced improvement of intracellular ROS but also prevented over-generated mitochondrial superoxide (Fig. 5B,D). It is suggested that CypA could increase intracellular ROS and mitochondrial superoxide induced by shikonin.

In order to investigate the relationship between CypA, intracellular ROS and mitochondrial superoxide, we introduced mitochondrial superoxide specific inhibitor MnTBAP. The results of fluorescence microscopy showed that the red fluorescence of the cells treated with shikonin was much brighter than that of the cells in the control group. In contrast, pretreatment with 40 μmol/L MnTBAP for 1 h significantly prevented the generation of mitochondrial superoxide induced by shikonin (Fig. 5E). Statistical analysis as well showed that CsA or MnTBAP pretreatment could inhibit the production of mitochondrial superoxide induced by shikonin (Fig. 5F). Moreover, shikonin-induced abnormal increases of intracellular ROS could be inhibited in the presence of CsA and MnTBAP (Fig. 5G,H). As an important transcription factor that regulates cellular oxidative stress response, NRF2(Nuclear Factor erythroid 2-Related Factor 2) reflects intracellular ROS levels. As shown in Fig. 5I, shikonin caused enhanced protein levels of NRF2 and p-NRF2, which could also be inhibited by CsA. These data indicated that CypA increased intracellular ROS via causing overproduction of mitochondrial superoxide.

### RIP1 and RIP3 upregulated the protein levels of CypA in cytoplasm, mitochondria and nucleus

**Figure 6:**
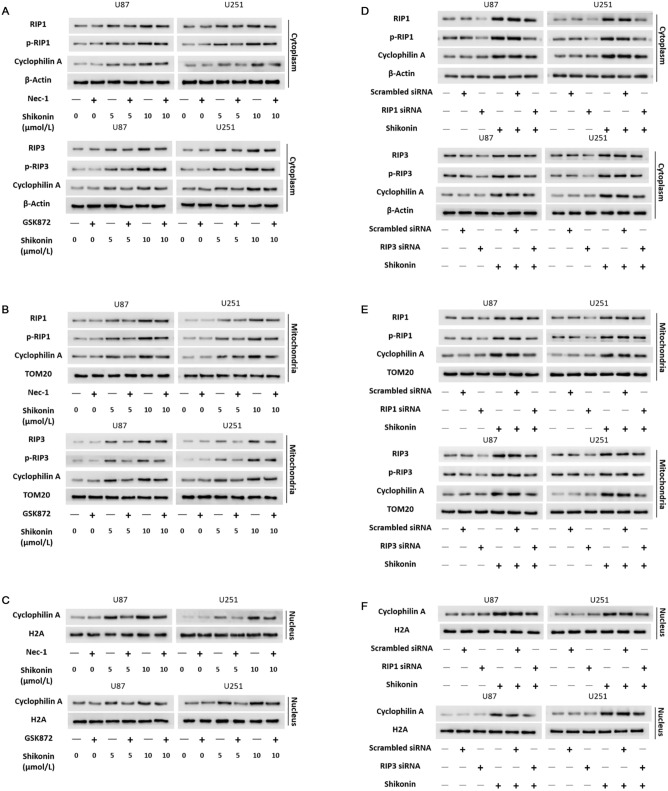
RIP1 and RIP3 upregulated the protein levels of CypA in cytoplasm, mitochondria and nucleus. (**A**–**C**) Western blotting analysis showed that inhibited RIP1 and RIP3 with Nec-1 and GSK872 prevented shikonin-induced up-regulation expression of CypA in cytoplasm, mitochondria and nucleus. (**D**–**F**) Knockdown of RIP1 and RIP3 with siRNA as well inhibited shikonin-induced up-regulation expression of CypA in cytoplasm, mitochondria and nucleus.

Considering that shikonin could induce the activation of RIP1 and RIP3, then the activated RIP1 and RIP3 induce the activation of downstream signaling during necroptosis^14^. Thus, we speculated RIP1 and RIP3 might regulate the activation of CypA during necroptosis induced by shikonin. As revealed by western blotting analysis, inhibited RIP1 and RIP3 with inhibitors Nec-1 and GSK872 could mitigate the up-regulation of CypA in cytoplasm, mitochondria and nucleus induced by shikonin at lower or higher dosages (Fig. 6A–C). Furthermore, knockdown of RIP1 and RIP3 with siRNA as well suppressed the expressional up-regulation of CypA caused by shikonin (Fig. 6D–F). Thus, these indicated that RIP1 and RIP3 regulated shikonin-induced activation of CypA in cytoplasm, mitochondria and nucleus.

### Shikonin induced CypA activation and chromatinolysis in glioma cells in vivo

**Figure 7:**
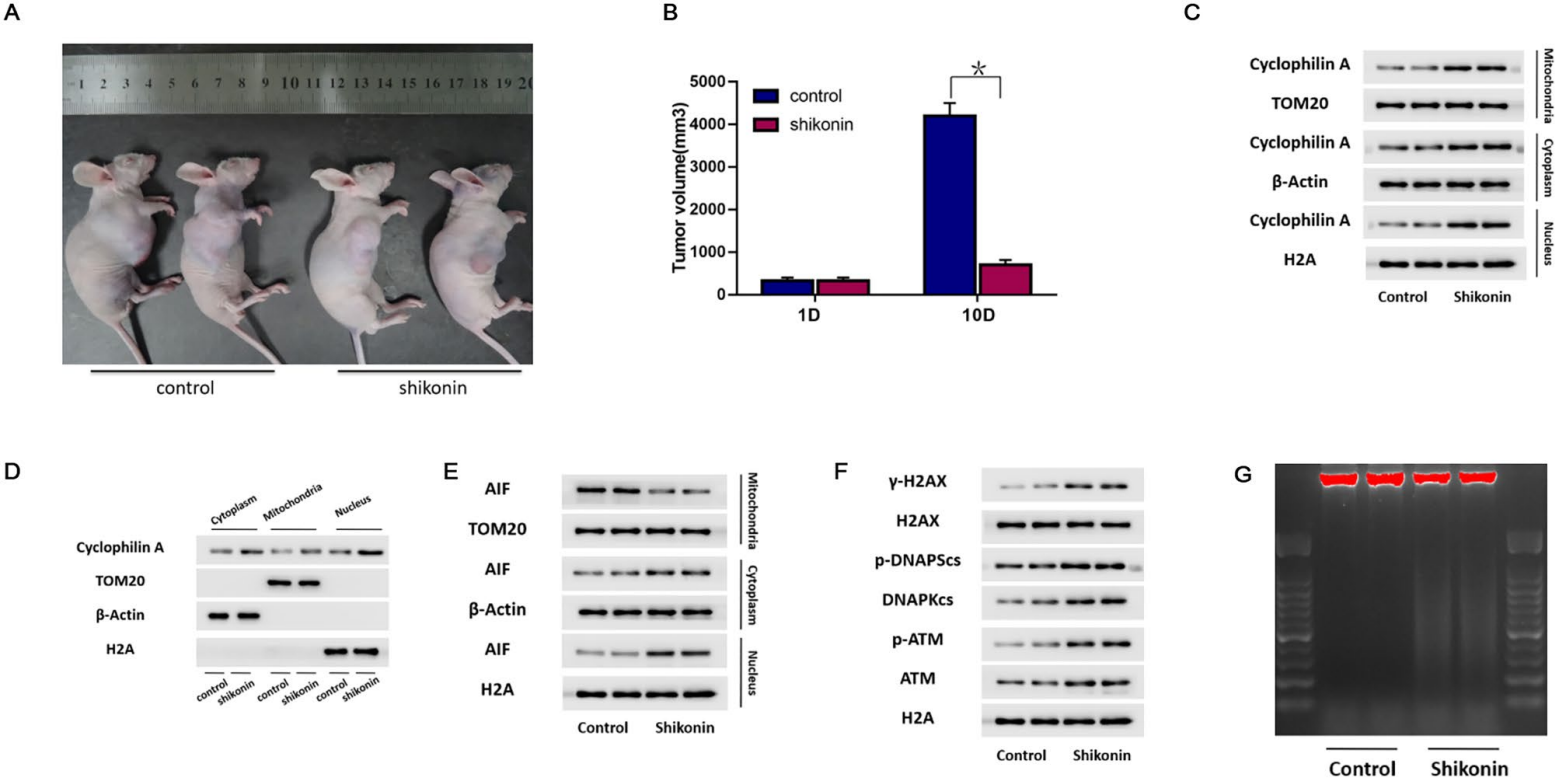
Shikonin induced CypA activation and chromatinolysis in glioma cells in vivo. (**A**) Representative images of the mice with xenografted glioma. (**B**) Shikonin treatment inhibited the growth of xenografted glioma. (**C**,**D**) Western blotting analysis showed that shikonin induced obvious up-regulation of CypA in cytoplasm, mitochondria and nucleus. (**E**) Shikonin improved both cytoplasmic and nuclear levels of AIF, but decreased mitochondrial AIF level. (**F**) Shikonin induced γ-H2AX formation and activation of ATM and DNAPKcs. (**G**) Agarose gel electrophoresis of the extracted nuclear DNA from the gliomas treated with or without shikonin showed that shikonin induced chromatinolysis in vivo. The values are expressed as mean ± SEM (n = 10 per group). *p < 0.01.

To test whether shikonin could induce CypA activation and chromatinolysis on glioma cells in vivo, C6 glioma cells were xenografted subcutaneously into the flank of nude mice as reported previously^14^. After being treated with shikonin at the dosage of 2 mg/kg once two days, the xenografted tumors decreased obviously in volumes when compared with those in control group (Fig. 7A). Statistical analysis of the tumor volumes proved as well that shikonin treatment effectively inhibited the growth of xenografted gliomas (Fig. 7B).

Then, we sacrificed the mice and isolated cytoplasm, nucleus and mitochondrion fractions by using differential centrifuge and analyzed the levels of target proteins in different fractions. As shown by western blotting analysis, CypA was induced to up-regulate by shikonin in cytoplasm, mitochondria and nucleus (Fig. 7C,D). This indicated that shikonin induced up-regulated expression of CypA activation and activated CypA accumulated at mitochondria and nuclear in vivo. We also found that shikonin treatment resulted in decreases in mitochondrial AIF, but improved cytoplasmic and nuclear levels of AIF (Fig. 7E). Moreover, nuclear levels of γ-H2AX, phospho-ATM and phospho-DNAPKcs were all up-regulated in shikonin-treated gliomas (Fig. 7F). Therefore, these indicated that shikonin induced nuclear translocation of AIF and γ-H2AX formation in glioma cells in vivo.

Furthermore, we conducted agarose gel electrophoresis of the extracted nuclear DNA from the removed gliomas. Consistently, it was showed that the genomic DNA extracted from shikonin-treated gliomas also presented smear band on agarose gel (Fig. 7G). Indicating that shikonin-induced chromatinolysis in vivo.

## Discussion

In this study, our results revealed for the first time that shikonin promotes time-dependent chromatinolysis and CypA activation. The activated CypA contributed to the AIF translocation to the nucleus and γ-H2AX formation. In vitro studies have shown that inhibition of CypA with its specific inhibitor CsA, or knockdown not only significantly mitigated shikonin-induced glioma cell death, but also prevented chromatinolysis. The mechanism involves the activated CypA to target the mitochondria, which then triggers the overproduction of mitochondrial superoxide, promotes AIF translocation into the nucleus by depolarizing the mitochondria, and intensifies the formation of γ-H2AX by promoting intracellular accumulation of ROS. Additionally, CypA in the nucleus can form a DNA degradation complex with AIF and γ-H2AX and participate in chromatinolysis. In contrast, inhibition of CypA through its specific inhibitor CsA or knocking it down using siRNA prevented overproduction of mitochondrial superoxide, nuclear translocation of AIF and formation of γ-H2AX, which effectively prevented chromatinolysis induced by shikonin. Furthermore, in the process of shikonin-induced necroptosis of the glioma cell, when specific inhibitors such as Nec-1 and GSK872 are used to inhibit RIP1 and RIP3, or with knockdown of RIP1 and RIP3 using siRNA, the expression of CypA can be inhibited; this proved that RIP1 and RIP3 can regulate CypA. Taken together, our results demonstrate that CypA contributes to shikonin-induced glioma cell necroptosis and promotion of chromatinolysis.

Chromatinolysis has been found to be involved in apoptosis and necrosis^22,23^. Transmission electron microscopy was considered the “gold standard” for cell death research, and is the most accurate method to distinguish apoptosis and necrosis. Under transmission electron microscope, the apoptotic cells showed clear condensed chromatin, membrane surface curl and apoptotic body formation^24,25^. Necrosis, which is different from apoptosis in the morphological features, is characterized by the swelling of organelles in the cytoplasm, loss of plasma membrane integrity, and condensation of chromatin into irregular fragments; however, the morphology of nucleus remains intact. In our previous study, the cells treated with shikonin did not appear as clumps of chromatin (a complex of DNA and histone protein) and the nucleus was electron-lucent with an intact nuclear membrane^13^. It is proved that shikonin induced necrosis in the glioma cells. Furthermore, in the process of apoptosis, DNA can be selectively cleaved into fragments of approximately 180-200 bp by endonuclease G in cells stressed with apoptosis inducers^17^. However, chromatinolysis was rapid and DNA was cleaved randomly in necrotic cells^26^. This can explain why the nuclear DNA extracted from apoptotic cell showed ladder bands after being electrophoresed agarose gel, while the nuclear DNA extracted from necrotic cells showed a continuous smear band. In this experiment, it was observed that shikonin induced a continuous smear band on the agarose gel and the smear band increased in a time-dependent manner. On the contrary, when using the CypA specific inhibitor CsA or knocking down CypA with siRNA, chromatinolysis and glioma cell death induced by shikonin were both alleviated. It is suggested that CypA can contribute to shikonin-induced glioma cell necroptosis and promotion of chromatinolysis.

Currently, the mechanism of necrotic chromatinolysis is still elusive, but many studies have shown that the nuclear translocation of AIF and the formation of γ-H2AX play an important role in promoting chromatinolysis^21^. As a flavoprotein is normally present in the mitochondrial inter-membrane space, AIF functioned as an endonuclease to degrade DNA after being truncated and redistributes into the nucleus^27^. In our study, after siRNA was used to knockdown AIF, the DNA fragmentation induced by shikonin was observed using agarose gel electrophoresis. Moreover, γ-H2AX can be used as an indicator of DNA DSBs both in vitro and in vivo. When DNA DSBs happens, γ-H2AX can be activated with the activation of ATM and DNAPKcs^9,10^. In addition, several evidences have shown that CypA is also involved in AIF nuclear translocation and it can positively regulate this process^27,28^. When AIF translocates into the nucleus, AIF interacts with CypA and phosphorylated H2AX through its C-terminal proline-rich module to form the DNA degradation complex; here, CypA plays the role of an endonuclease resulting in DNA degradation and necroptosis^6,11,12^. This may explain why in this study, when using an inhibitor CsA or siRNA to knockdown CypA, expression of AIF, γ-H2AX, p-ATM and other related proteins induced by shikonin was inhibited, and the cell death induced by shikonin was alleviated. In agarose gel electrophoresis, after the use of CsA and siRNA against CypA, the DNA fragmentation induced by shikonin was significantly relieved. In addition, in the neutral comet assay, the increases in the cells with comet tails and the improvement in DNA content in the comet tails induced by shikonin were both inhibited in the presence of CsA. This suggested that CypA participated in the regulation of shikonin-induced DNA DSBs in glioma cells.

Excessive production of ROS by oxidative stress will affect the function of cells. Therefore, inhibiting excessive production of ROS can effectively inhibit the occurrence of oxidative stress-mediated diseases and provide treatment. One of the greatest hazards of oxidative stress is DNA damage, especially DNA DSBs^29^. There is increasing evidence that excessive ROS can lead to nuclear translocation of AIF and formation of γ-H2AX, promoting chromatinolysis. In glioma cells treated with hydrogen peroxide, AIF was found to be released from mitochondria and translocated to the nucleus^14^. Shikonin can induce the overproduction of ROS in cells to cause DNA DSBs, lead to the activation of ATM and DNAPKcs, and promote the formation of γ-H2AX^30^. In this experiment, we found that ROS production induced by shikonin was affected by mitochondrial superoxide. After treatment with the mitochondrial superoxide inhibitor MnTBAP, the ROS content in cells was significantly reduced, the mitochondrial depolarization was significantly alleviated, and the shikonin-induced glioma cell death was inhibited. This indicated that shikonin promoted the death of glioma cells by overproducing mitochondrial superoxide by oxidative stress.

Some studies have shown that CypA can participate in the process of oxidative stress. Cao et al. reported that CypA can increase excessive ROS and promote oxidative stress in cardiomyocytes, and mediate inflammatory response^31,32^. Moreover, excessive ROS can promote the expression and activation of CypA at the same time^33^. Shikonin can improve the production of ROS in glioma cells in many ways^34^. In this study, we found for the first time that CypA is activated in shikonin-induced necroptosis in a time-dependent manner. The activated CypA can target mitochondrial and cause oxidative stress. The significant increase superoxide in the mitochondria leads to the depolarization of mitochondria and promotes the nuclear translocation of AIF. At the same time, the overproduction of superoxide in mitochondria can increase the content of intracellular ROS and aggravate the formation of γ-H2AX. The application of CsA or MnTBAP can significantly reduce the content of superoxide in mitochondria and intracellular ROS, inhibit nuclear translocation of AIF, and alleviate glioma cell death. This suggests that CypA plays an important role in the oxidative stress induced by shikonin.

Necroptosis can occur in colon cancer, non-small cell lung cancer and breast cancer via activation of RIP1 and RIP3^35–37^. In our previous studies, we demonstrated that shikonin induces glioma cell necroptosis in vitro by promoting RIP1/RIP3 necrosome formation^18^, which further contributed to shikonin-induced DNA DSBs and glycolysis suppression^14,15^. In this study, we confirmed that CypA can participate in the process of shikonin- induced necroptosis, and CypA is the downstream signal of RIP1 and RIP3. The protein expression of p-RIP1, p-RIP3 and CypA increased with the increase in shikonin concentration. CypA expression was inhibited when the specific inhibitors Nec-1 and GSK872 of RIP1 and RIP3 were used or when RIP1 and RIP3 were knocked down by siRNA. It is suggested that RIP1 and RIP3 can regulate the expression of CypA in the process of shikonin-induced necroptosis.

**Figure 8:**
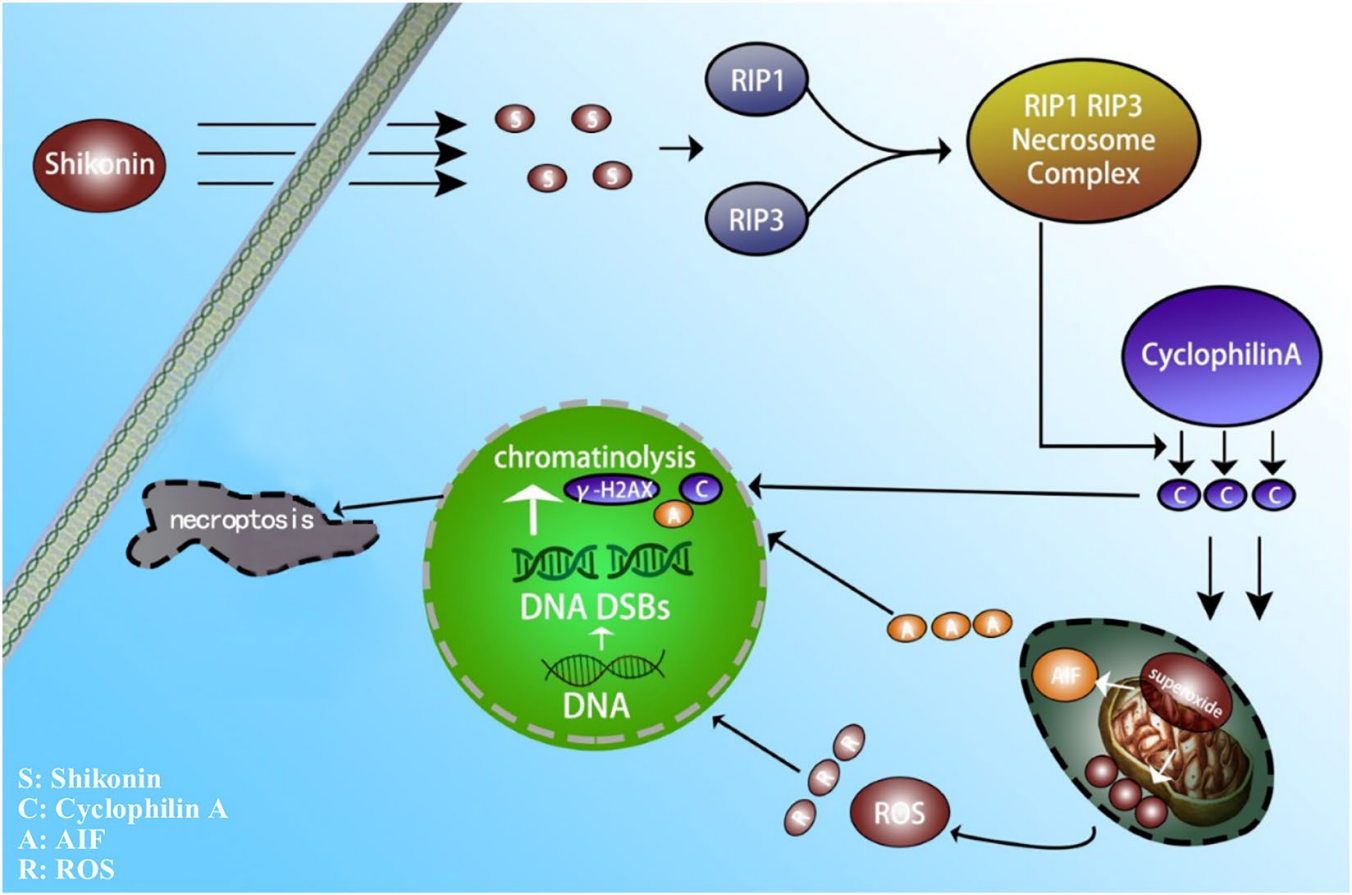
Schematic model for the role of CypA in shikonin-induced necroptosis and chromatinolysis. (Annotation S: Shikonin; C: Cyclophilin A; A: AIF; R: ROS).

In conclusion, we confirmed for the first time that CypA was involved in shikonin-induced necroptosis. Shikonin induced the activation of CypA in a time-dependent manner. The activated CypA can target mitochondria and trigger the excessive superoxide formation in the mitochondria, which leads to the depolarization of mitochondrial membrane potential, the release of AIF and nuclear translocation of AIF. At the same time, the overproduction of mitochondrial superoxide can increase intracellular ROS and aggravate the formation of γ-H2AX. CypA can also form a DNA degradation complex with AIF and γ-H2AX, which can cause chromatinolysis and promote glioma cell necroptosis (Fig. 8).

## Supplementary Information

Supplementary Information.

## Data Availability

All data generated or analysed during this study are included in this published article and its supplementary information files.
