# Supplementary material for: Cyclophilin A contributes to shikonin-induced glioma cell necroptosis and promotion of chromatinolysis
Source: Sci Rep. 2022 Aug 29;12:14675. doi: 10.1038/s41598-022-19066-y (PMC9424531; doi:10.1038/s41598-022-19066-y)
Supplement: Supplementary file 1 — Supplementary Information. [file 41598_2022_19066_MOESM1_ESM.pdf]

Cells death ratio after incubation with shikonin (%)

Time(min)<br>Cell lines	15	30	60	120
C6	6.60±1.50	13.05±2.10	21.53±2.00	33.05±2.31
SHG44	7.30±2.08	14.23±2.03	22.30±1.65	37.09±1.94
U87	6.94±1.83	13.79±1.61	22.36±1.61	33.14±2.45
U251	5.17±1.96	12.63±2.34	18.60±2.19	29.50±2.14
U373	5.87±1.79	11.94±2.12	20.51±1.65	32.34±2.14

U87 紫草素120mins cyclophilinA

cyclophilin A 18kd 1：1000 Abcam

U87 紫草素120mins actin

actin 42kd 1：1000 碧云天

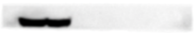

U87 紫草素120mins H2A

H2A 14kd 1：1000 Abcam

U87 紫草素120mins TOMM20

TOMM20 16kd 1：1000 Abcam

U251 紫草素120mins actin

actin 42kd 1：1000 碧云天

U251 紫草素120mins cyclophilinA

cyclophilin A 18kd 1：1000 Abcam

U251 紫草素120mins H2A

H2A 14kd 1：1000 Abcam

U251 紫草素120mins TOMM20

TOMM20 16kd 1：1000 Abcam

U87 紫草素时间梯度 cyclophilinA 胞核

cyclophilin A 18kd 1：1000 Abcam

U251 紫草素时间梯度 cyclophilinA 胞浆

cyclophilin A 18kd 1：1000 Abcam

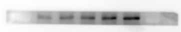

U87 紫草素时间梯度 cyclophilinA 胞浆

cyclophilin A 18kd 1：1000 Abcam

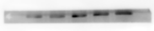

U251 紫草素时间梯度 H2A 胞核

H2A 14kd 1：1000 Abcam

U87 紫草素时间梯度 cyclophilinA 线粒体

cyclophilin A 18kd 1：1000 Abcam

U251 紫草素时间梯度 TOMM20 线粒体

TOMM20 16kd 1：1000 Abcam

U87 紫草素时间梯度 H2A 胞核

H2A 14kd 1：1000 Abcam

U251 紫草素时间梯度 cyclophilinA 线粒体

cyclophilin A 18kd 1：1000 Abcam

U87 紫草素时间梯度 TOMM20 线粒体

TOMM20 16kd 1：1000 Abcam

U87 紫草素时间梯度 actin 胞浆

actin 42kd 1：1000 碧云天

U251 紫草素时间梯度 actin 胞浆

actin 42kd 1：1000 碧云天

U251 紫草素时间梯度 cyclophilinA 胞核

cyclophilin A 18kd 1：1000 Abcam

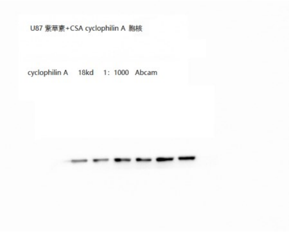
U87 紫草素+CSA cyclophilin A 胞核
cyclophilin A 18kd 1：1000 Abcam

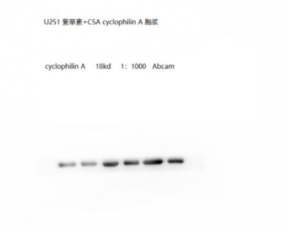
U251 紫草素+CSA cyclophilin A 胞浆
cyclophilin A 18kd 1：1000 Abcam

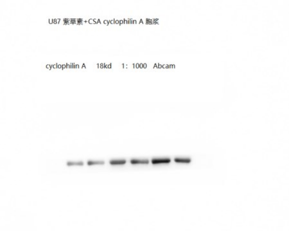
U87 紫草素+CSA cyclophilin A 胞浆
cyclophilin A 18kd 1：1000 Abcam

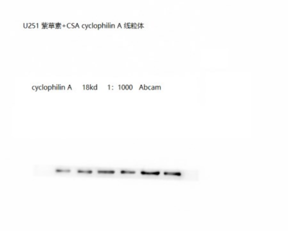
U251 紫草素+CSA cyclophilin A 线粒体
cyclophilin A 18kd 1：1000 Abcam

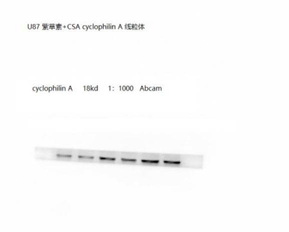
U87 紫草素+CSA cyclophilin A 线粒体
cyclophilin A 18kd 1：1000 Abcam

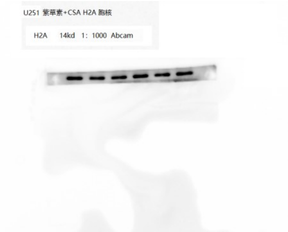
U251 紫草素+CSA H2A 胞核
H2A 14kd 1：1000 Abcam

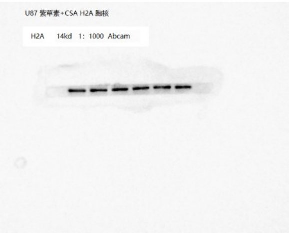
U87 紫草素+CSA H2A 胞核
H2A 14kd 1：1000 Abcam

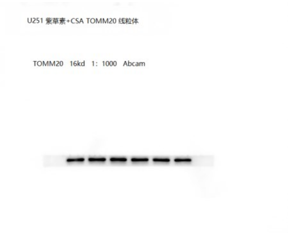
U251 紫草素+CSA TOMM20 线粒体
TOMM20 16kd 1：1000 Abcam

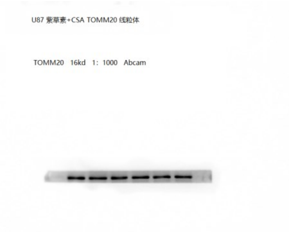
U87 紫草素+CSA TOMM20 线粒体
TOMM20 16kd 1：1000 Abcam

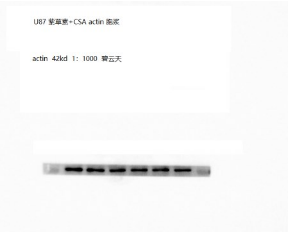
U87 紫草素+CSA actin 胞浆
actin 42kd 1：1000 碧云天

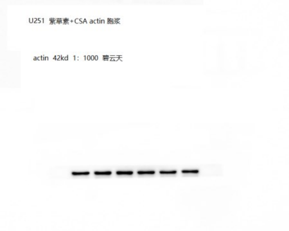
U251 紫草素+CSA actin 胞浆
actin 42kd 1：1000 碧云天

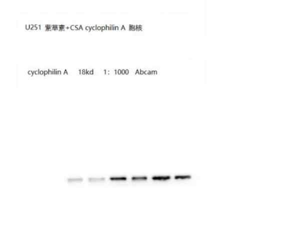
U251 紫草素+CSA cyclophilin A 胞核
cyclophilin A 18kd 1：1000 Abcam

U87 紫草素+cypA siRNA cylophilinA 胞核

cyclophilin A 18kd 1：1000 Abcam

U251 紫草素+cypA siRNA cylophilinA 胞浆

cyclophilin A 18kd 1：1000 Abcam

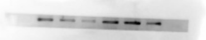

U87 紫草素+cypA siRNA cylophilinA 胞浆

cyclophilin A 18kd 1：1000 Abcam

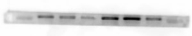

U251 紫草素+cypA siRNA cylophilinA 线粒体

cyclophilin A 18kd 1：1000 Abcam

U87 紫草素+cypA siRNA cylophilinA 线粒体

cyclophilin A 18kd 1：1000 Abcam

U251 紫草素+cypA siRNA H2A 胞核

H2A 14kd 1：1000 Abcam

U87 紫草素+cypA siRNA H2A 胞核

H2A 14kd 1：1000 Abcam

U251 紫草素+cypA siRNA TOMM20 线粒体

TOMM20 16kd 1：1000 Abcam

U87 紫草素+cypA siRNA TOMM20 线粒体

TOMM20 16kd 1：1000 Abcam

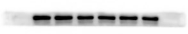

U87 紫草素+cypA siRNA actin 胞浆

actin 42kd 1：1000 碧云天

U251 紫草素+cypA siRNA actin 胞浆

actin 42kd 1：1000 碧云天

U251 紫草素+cypA siRNA cylophilinA 胞核

cyclophilin A 18kd 1：1000 Abcam

U251 紫草素120mins AIF

AIF 57，61kd 1:1000 Abcam

U87 紫草素120mins AIF

AIF 57，61kd 1:1000 Abcam

U87 紫草素时间梯度 AIF 胞浆

AIF 57，61kd 1:1000 Abcam

U87 紫草素时间梯度 AIF 线粒体

AIF 57，61kd 1:1000 Abcam

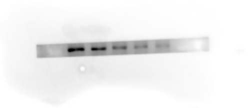

U251 紫草素时间梯度 AIF 胞核

AIF 57，61kd 1:1000 Abcam

U251 紫草素时间梯度 AIF 胞浆

AIF 57，61kd 1:1000 Abcam

U251 紫草素时间梯度 AIF 线粒体

AIF 57，61kd 1:1000 Abcam

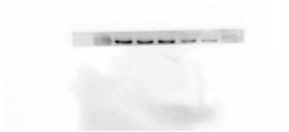

U87 紫草素时间梯度 AIF 胞核

AIF 57，61kd 1:1000 Abcam

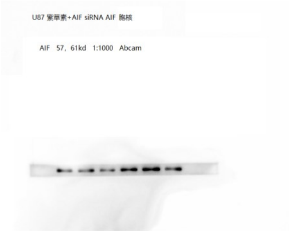
U87 紫草素+AIF siRNA AIF 胞核
AIF 57，61kd 1:1000 Abcam

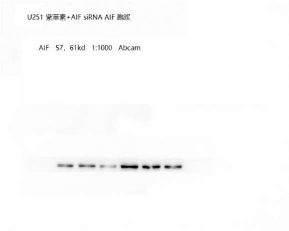
U251 紫草素+AIF siRNA AIF 胞浆
AIF 57，61kd 1:1000 Abcam

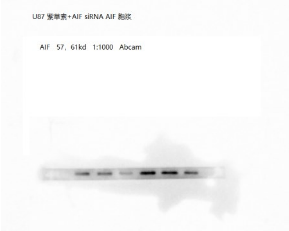
U87 紫草素+AIF siRNA AIF 胞浆
AIF 57，61kd 1:1000 Abcam

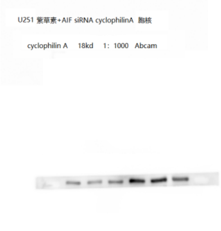
U251 紫草素+AIF siRNA cyclophilinA 胞核
cyclophilin A 18kd 1：1000 Abcam

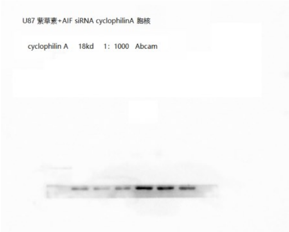
U87 紫草素+AIF siRNA cyclophilinA 胞核
cyclophilin A 18kd 1：1000 Abcam

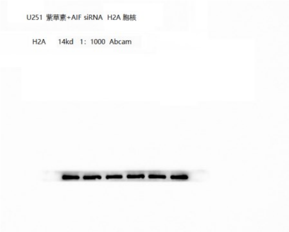
U251 紫草素+AIF siRNA H2A 胞核
H2A 14kd 1：1000 Abcam

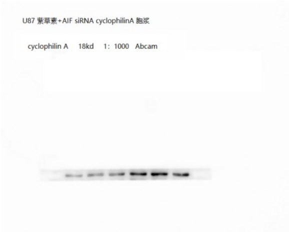
U87 紫草素+AIF siRNA cyclophilinA 胞浆
cyclophilin A 18kd 1：1000 Abcam

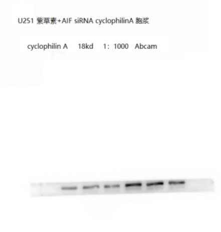
U251 紫草素+AIF siRNA cyclophilinA 胞浆
cyclophilin A 18kd 1：1000 Abcam

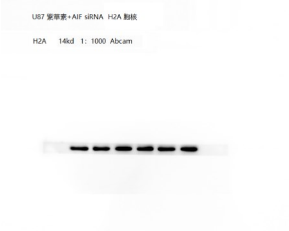
U87 紫草素+AIF siRNA H2A 胞核
H2A 14kd 1：1000 Abcam

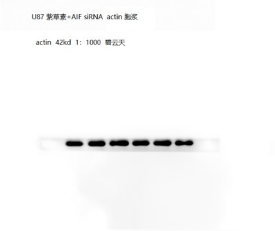
U87 紫草素+AIF siRNA actin 胞浆
actin 42kd 1：1000 碧云天

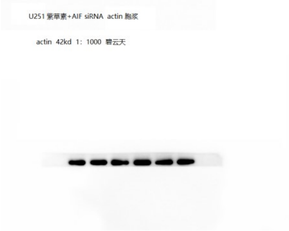
U251 紫草素+AIF siRNA actin 胞浆
actin 42kd 1：1000 碧云天

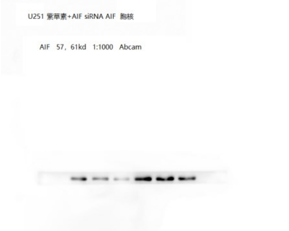
U251 紫草素+AIF siRNA AIF 胞核
AIF 57，61kd 1:1000 Abcam

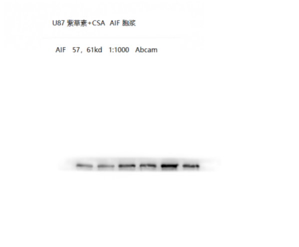
U87 紫草素+CSA AIF 胞浆
AIF 57，61kd 1:1000 Abcam

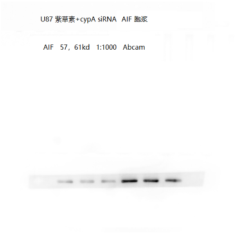
U87 紫草素+cypA siRNA AIF 胞浆
AIF 57，61kd 1:1000 Abcam

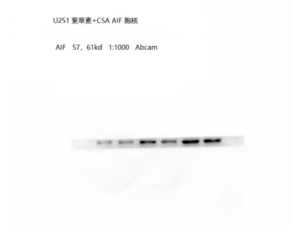
U251 紫草素+CSA AIF 胞核
AIF 57，61kd 1:1000 Abcam

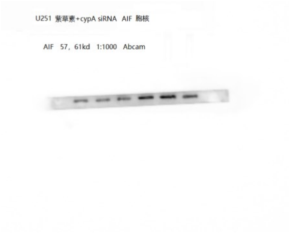
U251 紫草素+cypA siRNA AIF 胞核
AIF 57，61kd 1:1000 Abcam

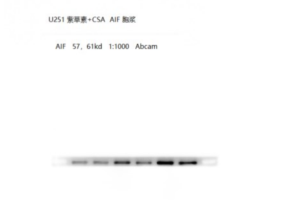
U251 紫草素+CSA AIF 胞浆
AIF 57，61kd 1:1000 Abcam

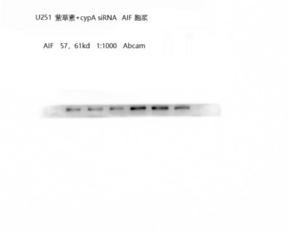
U251 紫草素+cypA siRNA AIF 胞浆
AIF 57，61kd 1:1000 Abcam

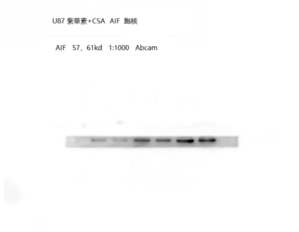
U87 紫草素+CSA AIF 胞核
AIF 57，61kd 1:1000 Abcam

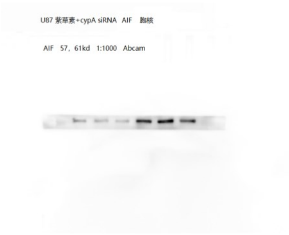
U87 紫草素+cypA siRNA AIF 胞核
AIF 57，61kd 1:1000 Abcam

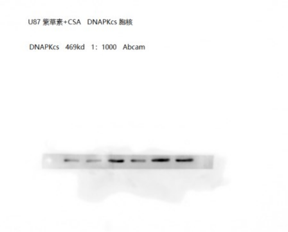
U87 紫草素+CSA DNAPKcs 胞核
DNAPKcs 469kd 1：1000 Abcam

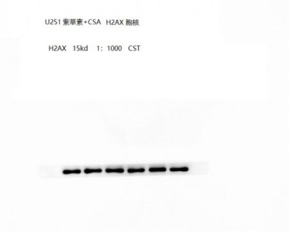
U251 紫草素+CSA H2AX 胞核
H2AX 15kd 1：1000 CST

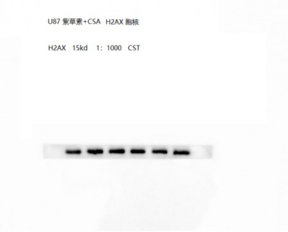
U87 紫草素+CSA H2AX 胞核
H2AX 15kd 1：1000 CST

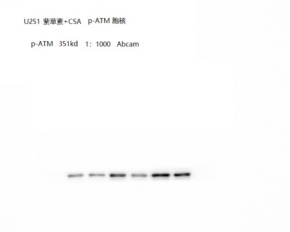
U251 紫草素+CSA p-ATM 胞核
p-ATM 351kd 1：1000 Abcam

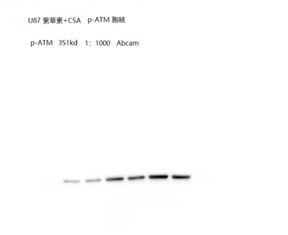
U87 紫草素+CSA p-ATM 胞核
p-ATM 351kd 1：1000 Abcam

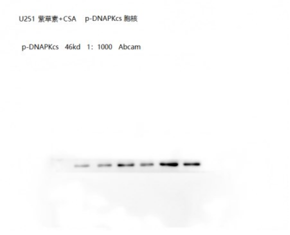
U251 紫草素+CSA p-DNAPKcs 胞核
p-DNAPKcs 46kd 1：1000 Abcam

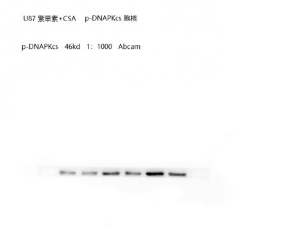
U87 紫草素+CSA p-DNAPKcs 胞核
p-DNAPKcs 46kd 1：1000 Abcam

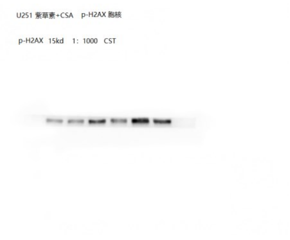
U251 紫草素+CSA p-H2AX 胞核
p-H2AX 15kd 1：1000 CST

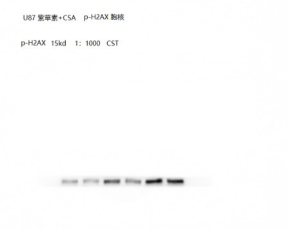
U87 紫草素+CSA p-H2AX 胞核
p-H2AX 15kd 1：1000 CST

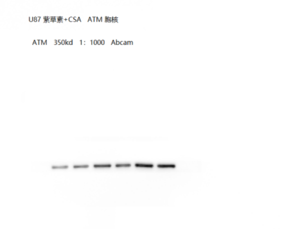
U87 紫草素+CSA ATM 胞核
ATM 350kd 1：1000 Abcam

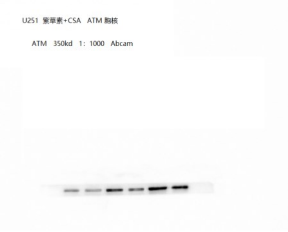
U251 紫草素+CSA ATM 胞核
ATM 350kd 1：1000 Abcam

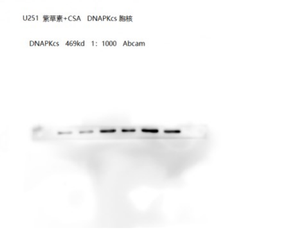
U251 紫草素+CSA DNAPKcs 胞核
DNAPKcs 469kd 1：1000 Abcam

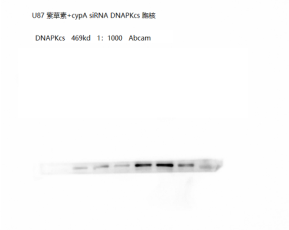
U87 紫草素+cypA siRNA DNAPKcs 胞核
DNAPKcs 469kd 1：1000 Abcam

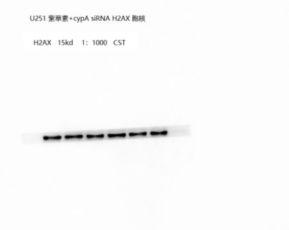
U251 紫草素+cypA siRNA H2AX 胞核
H2AX 15kd 1：1000 CST

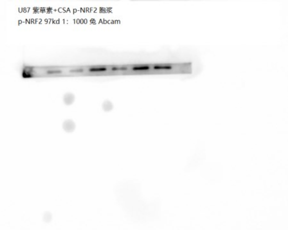
U87 紫草素+CSA p-NRF2 胞浆
p-NRF2 97kd 1：1000 兔 Abcam

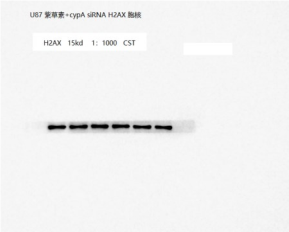
U87 紫草素+cypA siRNA H2AX 胞核
H2AX 15kd 1：1000 CST

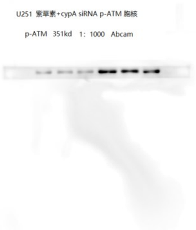
U251 紫草素+cypA siRNA p-ATM 胞核
p-ATM 351kd 1：1000 Abcam

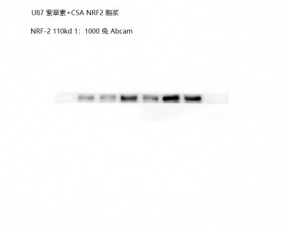
U87 紫草素+CSA NRF2 胞浆
NRF-2 110kd 1：1000 兔 Abcam

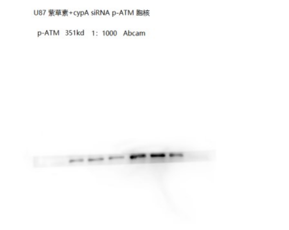
U87 紫草素+cypA siRNA p-ATM 胞核
p-ATM 351kd 1：1000 Abcam

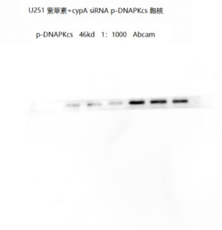
U251 紫草素+cypA siRNA p-DNAPKcs 胞核
p-DNAPKcs 46kd 1：1000 Abcam

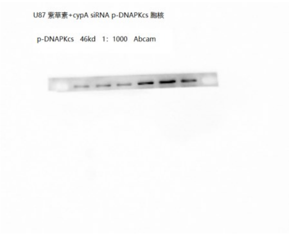
U87 紫草素+cypA siRNA p-DNAPKcs 胞核
p-DNAPKcs 46kd 1：1000 Abcam

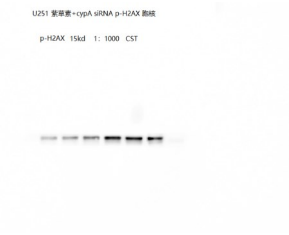
U251 紫草素+cypA siRNA p-H2AX 胞核
p-H2AX 15kd 1：1000 CST

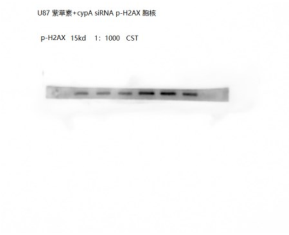
U87 紫草素+cypA siRNA p-H2AX 胞核
p-H2AX 15kd 1：1000 CST

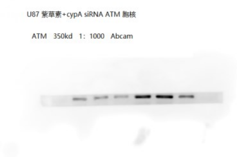
U87 紫草素+cypA siRNA ATM 胞核
ATM 350kd 1：1000 Abcam

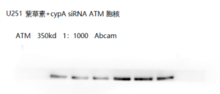
U251 紫草素+cypA siRNA ATM 胞核
ATM 350kd 1：1000 Abcam

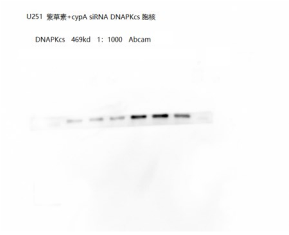
U251 紫草素+cypA siRNA DNAPKcs 胞核
DNAPKcs 469kd 1：1000 Abcam

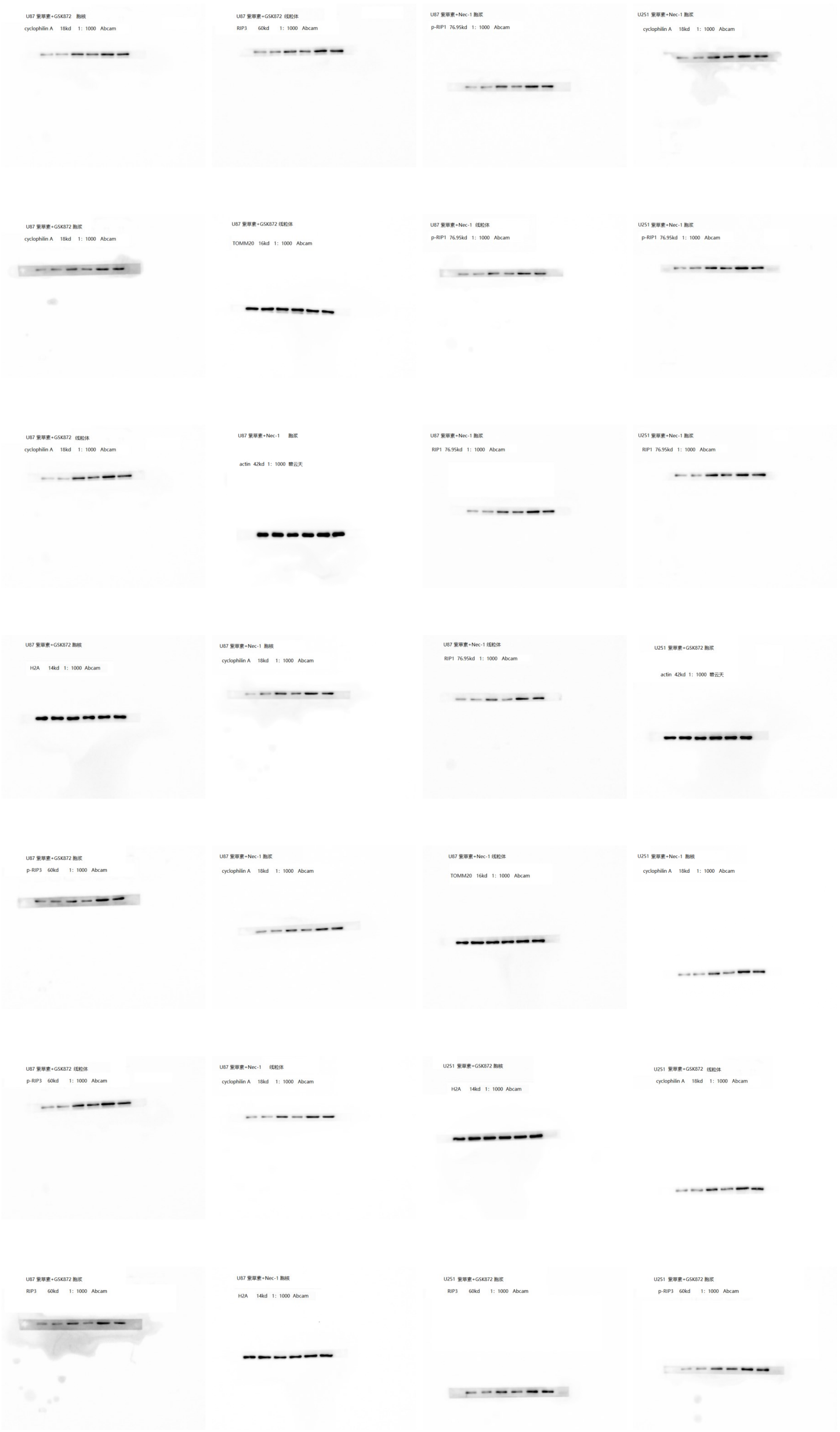

U87 紫草素+GSK872 胞核
cyclophilin A 18kd 1：1000 Abcam
U87 紫草素+GSK872 线粒体
RIP3 60kd 1：1000 Abcam
U87 紫草素+Nec-1 胞浆
p-RIP1 76.95kd 1：1000 Abcam
U251 紫草素+Nec-1 胞浆
cyclophilin A 18kd 1：1000 Abcam
U87 紫草素+GSK872 胞浆
cyclophilin A 18kd 1：1000 Abcam
U87 紫草素+GSK872 线粒体
TOMM20 16kd 1：1000 Abcam
U87 紫草素+Nec-1 线粒体
p-RIP1 76.95kd 1：1000 Abcam
U251 紫草素+Nec-1 胞浆
p-RIP1 76.95kd 1：1000 Abcam
U87 紫草素+GSK872 线粒体
cyclophilin A 18kd 1：1000 Abcam
U87 紫草素+Nec-1 胞浆
actin 42kd 1：1000 碧云天
U87 紫草素+Nec-1 胞浆
RIP1 76.95kd 1：1000 Abcam
U251 紫草素+Nec-1 胞浆
RIP1 76.95kd 1：1000 Abcam
U87 紫草素+GSK872 胞核
H2A 14kd 1：1000 Abcam
U87 紫草素+Nec-1 胞核
cyclophilin A 18kd 1：1000 Abcam
U87 紫草素+Nec-1 线粒体
RIP1 76.95kd 1：1000 Abcam
U251 紫草素+GSK872 胞浆
actin 42kd 1：1000 碧云天
U87 紫草素+GSK872 胞浆
p-RIP3 60kd 1：1000 Abcam
U87 紫草素+Nec-1 胞浆
cyclophilin A 18kd 1：1000 Abcam
U87 紫草素+Nec-1 线粒体
TOMM20 16kd 1：1000 Abcam
U251 紫草素+Nec-1 胞核
cyclophilin A 18kd 1：1000 Abcam
U87 紫草素+GSK872 线粒体
p-RIP3 60kd 1：1000 Abcam
U87 紫草素+Nec-1 线粒体
cyclophilin A 18kd 1：1000 Abcam
U251 紫草素+GSK872 胞核
H2A 14kd 1：1000 Abcam
U251 紫草素+GSK872 线粒体
cyclophilin A 18kd 1：1000 Abcam
U87 紫草素+GSK872 胞浆
RIP3 60kd 1：1000 Abcam
U87 紫草素+Nec-1 胞核
H2A 14kd 1：1000 Abcam
U251 紫草素+GSK872 胞浆
RIP3 60kd 1：1000 Abcam
U251 紫草素+GSK872 胞浆
p-RIP3 60kd 1：1000 Abcam

U251 紫草素+GSK872 线粒体

p-RIP3 60kd 1：1000 Abcam

U251 紫草素+Nec-1 胞核

H2A 14kd 1：1000 Abcam

U251 紫草素+GSK872 线粒体

RIP3 60kd 1：1000 Abcam

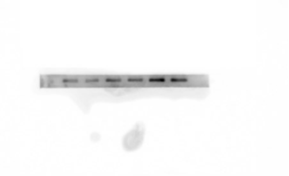

U251 紫草素+Nec-1 线粒体

p-RIP1 76.95kd 1：1000 Abcam

U251 紫草素+GSK872 线粒体

TOMM20 16kd 1：1000 Abcam

U251 紫草素+Nec-1 线粒体

RIP1 76.95kd 1：1000 Abcam

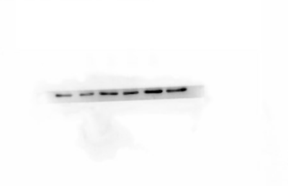

U251 紫草素+Nec-1 胞浆

actin 42kd 1：1000 碧云天

U251 紫草素+Nec-1 线粒体

TOMM20 16kd 1：1000 Abcam

U251 紫草素+Nec-1 胞核

cyclophilin A 18kd 1：1000 Abcam

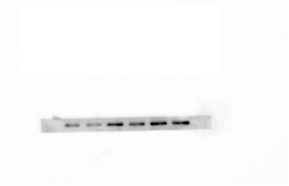

U87 紫草素+GSK872 胞浆

actin 42kd 1：1000 碧云天

U251 紫草素+GSK872 胞浆

cyclophilin A 18kd 1：1000 Abcam

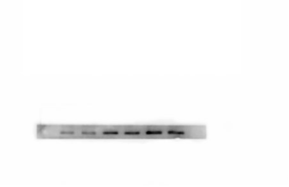

U251 紫草素+Nec-1 线粒体

cyclophilin A 18kd 1：1000 Abcam

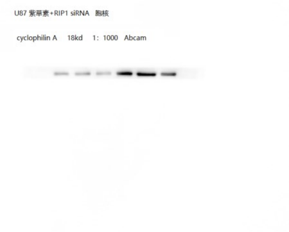
U87 紫草素+RIP1 siRNA 胞核
cyclophilin A 18kd 1：1000 Abcam
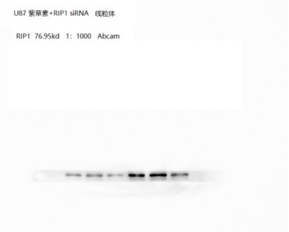
U87 紫草素+RIP1 siRNA 线粒体
RIP1 76.95kd 1：1000 Abcam
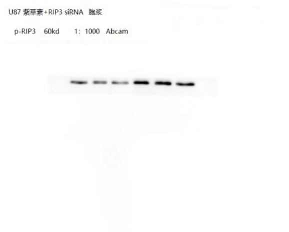
U87 紫草素+RIP3 siRNA 胞浆
p-RIP3 60kd 1：1000 Abcam
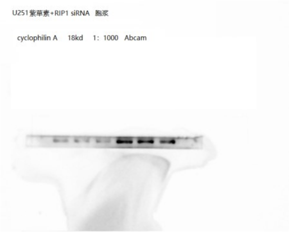
U251 紫草素+RIP1 siRNA 胞浆
cyclophilin A 18kd 1：1000 Abcam
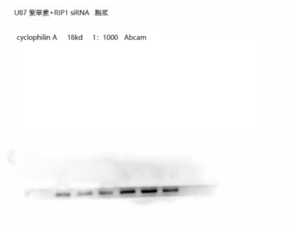
U87 紫草素+RIP1 siRNA 胞浆
cyclophilin A 18kd 1：1000 Abcam
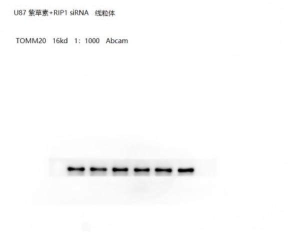
U87 紫草素+RIP1 siRNA 线粒体
TOMM20 16kd 1：1000 Abcam
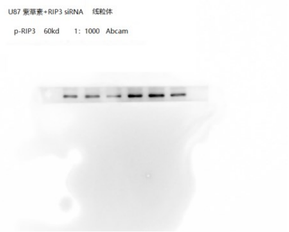
U87 紫草素+RIP3 siRNA 线粒体
p-RIP3 60kd 1：1000 Abcam
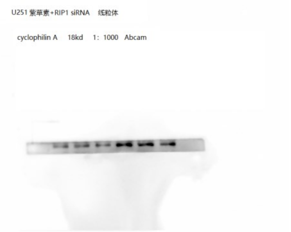
U251 紫草素+RIP1 siRNA 线粒体
cyclophilin A 18kd 1：1000 Abcam
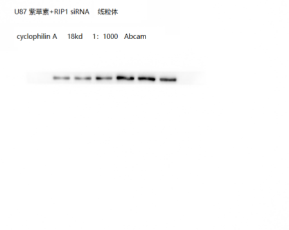
U87 紫草素+RIP1 siRNA 线粒体
cyclophilin A 18kd 1：1000 Abcam
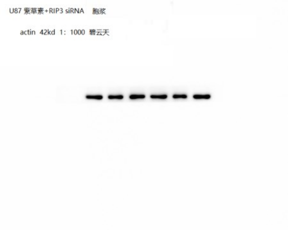
U87 紫草素+RIP3 siRNA 胞浆
actin 42kd 1：1000 碧云天
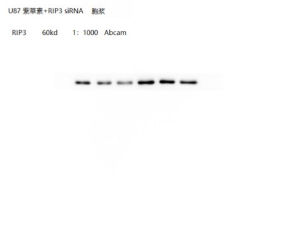
U87 紫草素+RIP3 siRNA 胞浆
RIP3 60kd 1：1000 Abcam
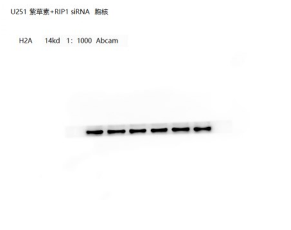
U251 紫草素+RIP1 siRNA 胞核
H2A 14kd 1：1000 Abcam
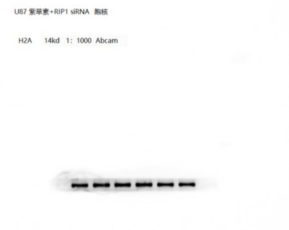
U87 紫草素+RIP1 siRNA 胞核
H2A 14kd 1：1000 Abcam
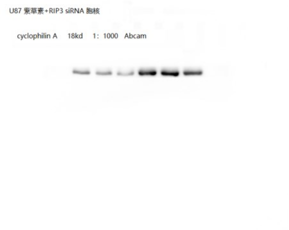
U87 紫草素+RIP3 siRNA 胞核
cyclophilin A 18kd 1：1000 Abcam
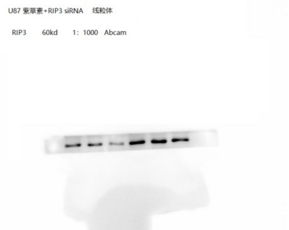
U87 紫草素+RIP3 siRNA 线粒体
RIP3 60kd 1：1000 Abcam
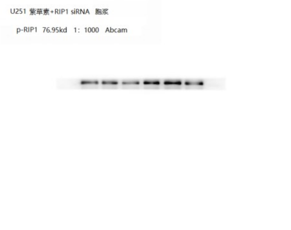
U251 紫草素+RIP1 siRNA 胞浆
p-RIP1 76.95kd 1：1000 Abcam
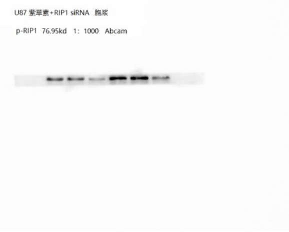
U87 紫草素+RIP1 siRNA 胞浆
p-RIP1 76.95kd 1：1000 Abcam
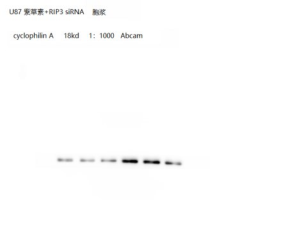
U87 紫草素+RIP3 siRNA 胞浆
cyclophilin A 18kd 1：1000 Abcam
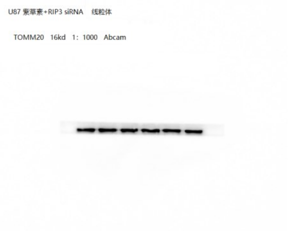
U87 紫草素+RIP3 siRNA 线粒体
TOMM20 16kd 1：1000 Abcam
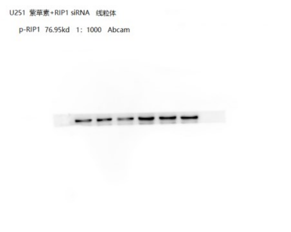
U251 紫草素+RIP1 siRNA 线粒体
p-RIP1 76.95kd 1：1000 Abcam
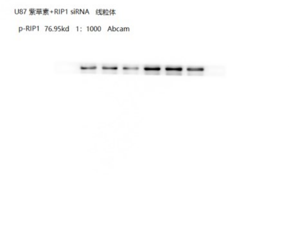
U87 紫草素+RIP1 siRNA 线粒体
p-RIP1 76.95kd 1：1000 Abcam
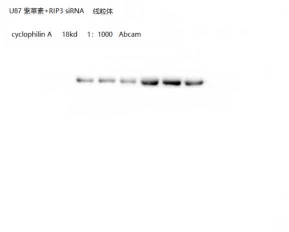
U87 紫草素+RIP3 siRNA 线粒体
cyclophilin A 18kd 1：1000 Abcam
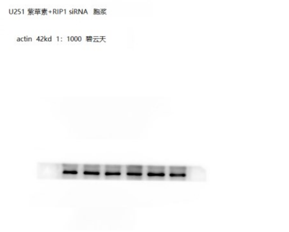
U251 紫草素+RIP1 siRNA 胞浆
actin 42kd 1：1000 碧云天
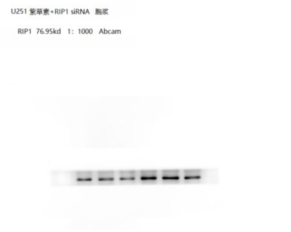
U251 紫草素+RIP1 siRNA 胞浆
RIP1 76.95kd 1：1000 Abcam
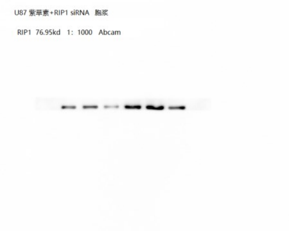
U87 紫草素+RIP1 siRNA 胞浆
RIP1 76.95kd 1：1000 Abcam
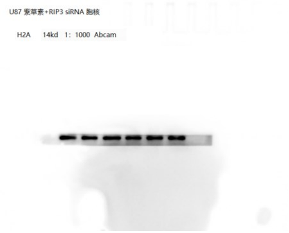
U87 紫草素+RIP3 siRNA 胞核
H2A 14kd 1：1000 Abcam
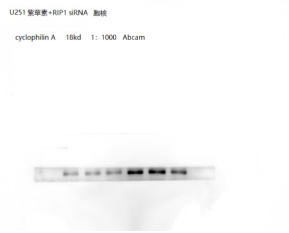
U251 紫草素+RIP1 siRNA 胞核
cyclophilin A 18kd 1：1000 Abcam
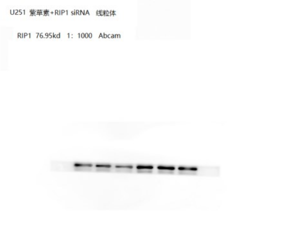
U251 紫草素+RIP1 siRNA 线粒体
RIP1 76.95kd 1：1000 Abcam

U251 紫草素+RIP1 siRNA 线粒体

TOMM20 16kd 1：1000 Abcam

U251 紫草素+RIP3 siRNA 线粒体

p-RIP3 60kd 1：1000 Abcam

U251 紫草素+RIP3 siRNA 胞浆

actin 42kd 1：1000 碧云天

U251 紫草素+RIP3 siRNA 胞浆

RIP3 60kd 1：1000 Abcam

U251 紫草素+RIP3 siRNA 胞核

cyclophilin A 18kd 1：1000 Abcam

U251 紫草素+RIP3 siRNA 线粒体

RIP3 60kd 1：1000 Abcam

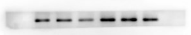

U251 紫草素+RIP3 siRNA 胞浆

cyclophilin A 18kd 1：1000 Abcam

U251 紫草素+RIP3 siRNA 线粒体

TOMM20 16kd 1：1000 Abcam

U251 紫草素+RIP3 siRNA 线粒体

cyclophilin A 18kd 1：1000 Abcam

U87 紫草素+RIP1 siRNA 胞浆

actin 42kd 1：1000 碧云天

U251 紫草素+RIP3 siRNA 胞核

H2A 14kd 1：1000 Abcam

U251 紫草素+RIP3 siRNA 胞浆

p-RIP3 60kd 1：1000 Abcam

裸鼠 胞浆

actin 42kd 1：1000 碧云天

裸鼠 胞浆

cyclophilin A 18kd 1：1000 Abcam

裸鼠 胞核

p-DNAPKcs 46kd 1：1000 Abcam

裸鼠 胞核

AIF 57，61kd 1:1000 Abcam

裸鼠 线粒体

cyclophilin A 18kd 1：1000 Abcam

裸鼠 胞核

p-H2AX 15kd 1：1000 CST

裸鼠 胞浆

AIF 57，61kd 1:1000 Abcam

裸鼠 胞核

DNAPKcs 469kd 1：1000 Abcam

裸鼠 胞浆 线粒体 胞核

TOMM20 16kd 1：1000 Abcam

裸鼠 线粒体

AIF 57，61kd 1:1000 Abcam

裸鼠 胞核

H2A 14kd 1：1000 Abcam

裸鼠 线粒体

TOMM20 16kd 1：1000 Abcam

裸鼠 胞核

ATM 350kd 1：1000 Abcam

裸鼠 胞浆 线粒体 胞核

H2A 14kd 1：1000 Abcam

裸鼠 胞浆 线粒体 胞核

actin 42kd 1：1000 碧云天

裸鼠 胞核

cyclophilin A 18kd 1：1000 Abcam

裸鼠 胞核

H2AX 15kd 1：1000 CST

裸鼠 胞浆 线粒体 胞核

cyclophilin A 18kd 1：1000 Abcam

裸鼠 胞核

p-ATM 351kd 1：1000 Abcam
